# The coordinating contracts of supply chain in a fuzzy decision environment

**DOI:** 10.1186/s40064-016-2401-4

**Published:** 2016-07-01

**Authors:** Shengju Sang

**Affiliations:** Department of Economics and Management, Heze University, Heze, 274015 China

**Keywords:** Supply chain coordination, Revenue sharing contract, Return contract, Fuzzy set, Triangular fuzzy number, 90B05, 90C70

## Abstract

The rapid change of the product life cycle is making the parameters of the supply chain models more and more uncertain. Therefore, we consider the coordination mechanisms between one manufacturer and one retailer in a fuzzy decision marking environment, where the parameters of the models can be forecasted and expressed as the triangular fuzzy variables. The centralized decision-making system, two types of supply chain contracts, namely, the revenue sharing contract and the return contract are proposed. To obtain their optimal policies, the fuzzy set theory is adopted to solve these fuzzy models. Finally, three numerical examples are provided to analyze the impacts of the fuzziness of the market demand, retail price and salvage value of the product on the optimal solutions in two contracts. It shows that in order to obtain more fuzzy expected profits the retailer and the manufacturer should seek as low fuzziness of demand, high fuzziness of the retail price and the salvage value as possible in both contracts.

## Background

In recent years, supply chain coordination mechanisms such as the revenue sharing contract and return contract have become the hot topics faced by both some scholars and practitioners. Coordination among the supply chain members is an important strategy in supply chain. Contracts are considered as effective instruments to bring the manufacturer and the retailer in a decentralized supply chain to operate in coordination. The contract can overcome the problem of double marginalization exited in supply chain system. The retailer can be induced to order right quantity, and maximize the total profit of the supply chain system through the negotiation among the members with contracts. In such supply chain contracts, the risks caused by the uncertain demand are shifted from the retailers to the manufacturers. In return, the order quantities of the retailers in such supply chain contracts are equal to those in the centralized decision making system so as to maximize the sum of profits for the manufacturers and retailers.

Revenue sharing (RS) contract has achieved a great success in practice among the supply chain members and widely be used in the movie industry. Cachon and Lariviere (2005) compared the RS contract to some other kinds of contracts and pointed out its strengths and limitations. Chen et al. (2011) studied the RS contract in a two stage supply chain, where the demand was a function of retail price and size of self-space. Rhee et al. (2010, 2014) used the spanning RS contract for coordinating a multi-echelon supply chain in a random demand setting, where the most downstream member shared his profit with all the other members. Krishnan and Winter (2011) examined the coordinating role of the RS contract comprising two competitive retailers and one manufacturer. Zhang et al. (2012) also studied the RS contract problem with two competitive retailers in a demand disruption setting. Recently, Palsule-Desai (2013) proposed a revenue-dependent RS contract to coordinate the supply chain in a two-period model. Giovanni (2014) proposed a reverse RS contract to coordinate the closed-loop supply chain with green advertising strategies. Panda (2014) used the RS contract aimed at coordinating the socially responsible issues of supply chain with a linear demand. Hsueh (2014) also developed a RS contract with corporate social responsibility in a random demand environment.

Return contract is also widely adopted for some high new products with short life cycles in a number of industries such as personal computers, fashion apparel and toys. Yao et al. (2005) discussed the impact of information sharing on the return contract in both traditional retail channels and direct channels. Chen (2011) also studied the impact of the sharing customer returns information on the return policy in the Manufacturer Stackelberg game scenario. Yao et al. (2008) examined the effects of price-sensitivity factors on the return contract in a stochastic and price-dependent demand setting. Zhao et al. (2014) also studied the return contract in a price-dependent downward-sloping demand environment, but they mainly focused on the problem of the demand uncertainty level. Chen and Bell (2011) used the return policy for coordinating a decentralized supply chain in a price-dependent demand setting. Chen and Bell (2012) also examined the coordination issue of the dual-channel through return policies. In addition, Ai et al. (2012) proposed a full return policy to study the competition problem among two supply chains with uncertain demand. Wu (2013) also used a return contract to coordinate the competing supply chains, where two power distributions namely Vertical Integration model and Manufacturer’s Stackelberg game were considered. Huang et al. (2014) developed a return contract to coordinate the supply chain comprising many retailers with a secondary market. Yoo (2014) used a return policy to coordinate the supply chain, where the supplier has two different risk attitudes: risk averse and risk neutral.

The works mentioned above analyzed the revenue sharing contract and the return contract in a linear or random market demand. In recent years, fuzzy set theory has been adopted by more and more scholars to solve the problems in the supply chain management. For instance, Xu and Zhai (2008) considered the demand as a triangular fuzzy variable and developed a fuzzy newsboy model. Xu and Zhai (2010) also extend their work to the coordination problem, where the demand was considered as an L-R fuzzy number. Hu et al. (2010) developed the fuzzy decentralized and centralized decision marking systems with imperfect quality in a fuzzy random demand setting. Kazemi et al. (2010) investigated the EOQ model with backorders in a fuzzy decision environment, where the parameters of the model were considered as the trapezoidal and triangular numbers. Samal and Pratihar (2014) also studied the fuzzy EOQ model without and with backordering. In addition, Chen and Cheng (2014) studied the inventory policies with a multi-stage supply chain, where the demand and inventory cost were considered as fuzzy variables. Ye and Li (2011) developed a Stackelberg game model between a supplier and a retailer under a fuzzy market demand environment, where the risks of the supply chain members were considered. Yu and Jin (2011) adopted signed distance method to study the return contract under uncertain demands, where the demand and the retail price were regarded as the triangular fuzzy numbers. Yu et al. (2013) also studied the fuzzy newsboy model in a price-dependent demand environment. In recent paper, Chang and Yeh (2013) analyzed the centralized and decentralized decision-making systems with returnable products, where the demand was considered as the trapezoidal number. Sang (2013) studied the revenue sharing contract and the return contract comprising multiple competing retailers with fuzzy demand. Zhang et al. (2014) adopted the crisp possibilistic mean method to study a two-level return contract in a fuzzy random demand environment. Recently, some researchers such as Boutkhoum et al. (2015), Abdolmajidi et al. (2016) and Hanine et al. (2016) studied the applications of fuzzy theory in other optimal problems. Boutkhoum et al. (2015) studied the industrial location selection problem in a fuzzy decision making environment. Hanine et al. (2016) proposed the fuzzy TODIM and fuzzy AHP methods to select the landfill location. Abdolmajidi et al. (2016) used fuzzy logic for modeling the spatial data infrastructure development.

To the best of our knowledge, there is no study on the RS contract and the return contract that the parameters of supply chain models are all characterize by the fuzzy variables. However, in the real word, the rapid change of the product life cycle makes the parameters of the supply chain models more and more uncertain. These uncertainties may be the retail price, market demand, costs of the supply chain members, etc. For practical purpose, the linguistic terms are usually used to describe these uncertainties, such as “the retail price is about *b*, but definitely not greater than *c* and not less than *a*”. Therefore, we assume the fuzzy number variables can be forecasted and expressed as triangular membership functions. Triangular fuzzy numbers are easy to handle arithmetically and have intuitive interpretations. Triangular fuzzy numbers as one types of left–right fuzzy numbers are adopted because they are considered the most fit for modeling uncertain parameters (Xu and Zhai 2008; Yu et al. 2013).

This study aims at developing coordination mechanisms of the manufacturer and the retailer and pursuing their optimal strategies when the parameters of the models are fuzzy. The contributions of this article are as follows. Firstly, we study the supply chain coordination mechanisms in a fuzzy decision making environment. The market demand, retail price, costs of the manufacturer and the retailer, savage value of the unsold product are all fuzziness. Secondly, both the RS contract and the return contract are considered in a fuzzy decision making environment. Thirdly, we discuss the impacts of the fuzziness of the market demand, retail price, and savage value of the product on the optimal policies in two contracts. These can improve decision making of the experts in supply chain management.

The article is organized as follows. Some definitions and propositions about the triangular fuzzy number related to this paper are introduced in section “Preliminaries”. The notations and assumptions of the models are introduced in section “Notations and assumptions”. In section “Models and solution approaches”, the centralized decision-making system, the RS contract and the return contract in a fuzzy decision making environment are proposed. In section “Numerical examples”, some numerical examples are given to elucidate the solutions of each model. Last section “Conclusions” summarizes the work.

## Preliminaries

In this section, we introduce some definitions and propositions about the fuzzy set theory for modeling the supply chain contract with uncertain factors.

### **Definition 1**

The fuzzy set $\tilde{A} = \left( {a_{1} ,a_{2} ,a_{3} } \right)$ is said to be the triangular fuzzy number, when it has a following membership function

1 $$\mu_{{\tilde{A}}} \left( x \right) = \left\{ {\begin{array}{*{20}l} {\frac{{x - a_{1} }}{{a_{2} - a_{1} }},} \hfill &\quad {x \in \left[ {a_{1} ,a_{2} } \right],} \hfill \\ {\frac{{a_{3} - x}}{{a_{3} - a_{2} }},} \hfill &\quad {x \in \left( {a_{2} ,a_{3} } \right],} \hfill \\ {0,} \hfill & \quad{\text{otherwise} .} \hfill \\ \end{array} } \right.$$

where $a_{1}$, $a_{2}$ and $a_{3}$ are crisp numbers with $a_{1} < a_{2} < a_{3}$. When $x \in \left[ {a_{1} ,a_{2} } \right]$, $\tilde{A}_{L} \left( x \right) = \frac{{x - a_{1} }}{{a_{2} - a_{1} }}$ is continuous and increasing about $x$. When $x \in \left( {a_{2} } \right.\left. {a_{3} } \right]$, $\tilde{A}_{R} \left( x \right) = \frac{{a_{3} - x}}{{a_{3} - a_{2} }}$ is continuous and decreasing about $x$.

### **Definition 2**

If $a_{1} > 0$, then $\tilde{A} = \left( {a_{1} ,a_{2} ,a_{3} } \right)$ is called the positive triangular fuzzy number.

### **Definition 3**

Given $\alpha \in \left[ {0,1} \right]$, the set $\tilde{A}\left( \alpha \right) = \left( {x|\mu_{{\tilde{A}}} \left( x \right) \ge \alpha } \right)$ is called the $\alpha$ cut set of $\tilde{A}$ and is denoted by the interval $t[ {\tilde{A}_{L}^{ - 1} ( \alpha ),\tilde{A}_{R}^{ - 1} ( \alpha )} ]$, with

2 $$\tilde{A}_{L}^{ - 1} \left( \alpha \right) = \inf \left\{ {x \in R:\;\mu_{{\tilde{A}(x)}} \ge \alpha } \right\},\quad {\text{and}}\quad \tilde{A}_{R}^{ - 1} \left( \alpha \right) = \sup \left\{ {x \in R:\;\mu_{{\tilde{A}(x)}} \ge \alpha } \right\}.$$

where $\tilde{A}_{L}^{ - 1} \left( \alpha \right)$ and $\tilde{A}_{R}^{ - 1} \left( \alpha \right)$ are the left and right cut sets of $\tilde{A}\left( \alpha \right)$.

### *Example 1*

Given $\alpha \in \left[ {0,1} \right]$, the $\alpha$ cut set of $\tilde{A} = \left( {a_{1} ,a_{2} ,a_{3} } \right)$ can be given as

3 $$\tilde{A}_{L}^{ - 1} \left( \alpha \right) = \left( {1 - \alpha } \right)a_{1} + \alpha a_{2} ,\quad {\text{and}}\quad \tilde{A}_{R}^{ - 1} \left( \alpha \right) = \alpha a_{2} + \left( {1 - \alpha } \right)a_{3} .$$

where $\tilde{A}_{L}^{ - 1}$ and $\tilde{A}_{R}^{ - 1}$ are the inverse functions of $\tilde{A}_{L}$ and $\tilde{A}_{R}$ respectively.

The following Propositions 1 and 2 can be obtained through the extension principle of the fuzzy set theory.

### **Proposition 1**

*Given*$\alpha \in \left[ {0,1} \right]$*, let*$\tilde{A}$*be a positive triangular fuzzy variable with*$\alpha$*cut set*$[ {\tilde{A}_{L}^{ - 1} ( \alpha),\tilde{A}_{R}^{ - 1} ( \alpha )} ]$*, then*

4 $$k\tilde{A}\left( \alpha \right) = \left\{ \begin{aligned} \left[ {k\tilde{A}_{L}^{ - 1} \left( \alpha \right),k\tilde{A}_{R}^{ - 1} \left( \alpha \right)} \right],\;\;k \in R^{ + } , \hfill \\ \left[ {k\tilde{A}_{R}^{ - 1} \left( \alpha \right),k\tilde{A}_{L}^{ - 1} \left( \alpha \right)} \right],\;\;k \in R^{ - } . \hfill \\ \end{aligned} \right.$$

### **Proposition 2**

*Given*$\alpha \in \left[ {0,1} \right]$*, let*$\tilde{B}$*and*$\tilde{C}$*be two positive triangular fuzzy numbers with*$\alpha$*cut sets*$[ {\tilde{B}_{L}^{ - 1} ( \alpha ),\tilde{B}_{R}^{ - 1} ( \alpha )} ]$*and*$[ {\tilde{C}_{L}^{ - 1} ( \alpha ),\tilde{C}_{R}^{ - 1} ( \alpha)}]$*, respectively. Then we have*

$$({\text{a}})\quad \tilde{B}\left( \alpha \right) + \tilde{C}\left( \alpha \right) = \left[ {\tilde{B}_{L}^{ - 1} \left( \alpha \right) + \tilde{C}_{L}^{ - 1} \left( \alpha \right),\tilde{B}_{R}^{ - 1} \left( \alpha \right) + \tilde{C}_{R}^{ - 1} \left( \alpha \right)} \right],$$

$$({\text{b}})\quad \tilde{B}\left( \alpha \right) - \tilde{C}\left( \alpha \right) = \left[ {\tilde{B}_{L}^{ - 1} \left( \alpha \right) - \tilde{C}_{R}^{ - 1} \left( \alpha \right),\tilde{B}_{R}^{ - 1} \left( \alpha \right) - \tilde{C}_{L}^{ - 1} \left( \alpha \right)} \right],$$

$$({\text{c}})\quad \left( {\tilde{A}\tilde{B}} \right)_{L}^{ - 1} \left( \alpha \right) = \tilde{A}_{L}^{ - 1} \left( \alpha \right)\tilde{B}_{L}^{ - 1} \left( \alpha \right),$$

5 $$({\text{d}})\quad \left( {\tilde{A}\tilde{B}} \right)_{R}^{ - 1} \left( \alpha \right) = \tilde{A}_{R}^{ - 1} \left( \alpha \right)\tilde{B}_{R}^{ - 1} \left( \alpha \right).$$

In addition, to solve the fuzzy optimal models, we must first convert the fuzzy number into a crisp one. Our study adopts the ranking method proposed by Liu and Liu (2002), which is given by

### **Proposition 3**

(Liu and Liu 2002) *Let*$\tilde{A}$*be a positive triangular number with*$\alpha$*cut set*$\left[ {\tilde{A}_{L}^{ - 1} \left( \alpha \right),\tilde{A}_{R}^{ - 1} \left( \alpha \right)} \right]$*, then the expected value*$E\left[ {\tilde{A}} \right]$*is*

6 $$E\left[ {\tilde{A}} \right] = \frac{1}{2}\int_{{{\kern 1pt} 0}}^{{{\kern 1pt} 1}} {\left[ {\tilde{A}_{L}^{ - 1} \left( \alpha \right) + \tilde{A}_{R}^{ - 1} \left( \alpha \right)} \right]} \text{d} \alpha .$$

### *Example 2*

The triangular fuzzy number $\tilde{A} = \left( {a_{1} ,a_{2} ,a_{3} } \right)$ has an expected value

7 $$E\left[ {\tilde{A}} \right] = \frac{1}{2}\int_{{{\kern 1pt} 0}}^{{{\kern 1pt} 1}} {\left[ {\tilde{A}_{L}^{ - 1} \left( \alpha \right) + \tilde{A}_{R}^{ - 1} \left( \alpha \right)} \right]} \text{d} \alpha = \frac{1}{2}\int_{{{\kern 1pt} 0}}^{{{\kern 1pt} 1}} {\left[ {\left( {1 - \alpha } \right)a_{1} + \alpha a_{2} + \alpha a_{2} + \left( {1 - \alpha } \right)a_{3} } \right]\text{d} \alpha } = \frac{{a_{1} + 2a_{2} + a_{3} }}{4}$$

### *Example 3*

For two nonnegative independent triangular fuzzy numbers $\tilde{A} = \left( {a_{1} ,a_{2} ,a_{3} } \right)$ and $\tilde{B} = \left( {b_{1} ,b_{2} ,b_{3} } \right)$, we have

8 $$\begin{aligned} E\left[ {\tilde{A}\tilde{B}} \right] & = \frac{1}{2}\int_{{{\kern 1pt} 0}}^{{{\kern 1pt} 1}} {\left[ {\left( {\tilde{A}\tilde{B}} \right)_{L}^{ - 1} \left( \alpha \right) + \left( {\tilde{A}\tilde{B}} \right)_{R}^{ - 1} \left( \alpha \right)} \right]} \text{d} \alpha \\ & = \frac{1}{2}\int_{{{\kern 1pt} 0}}^{{{\kern 1pt} 1}} {\left[ {\tilde{A}_{L}^{ - 1} \left( \alpha \right)\tilde{B}_{L}^{ - 1} \left( \alpha \right) + \tilde{A}_{R}^{ - 1} \left( \alpha \right)\tilde{B}_{R}^{ - 1} \left( \alpha \right)} \right]} \text{d} \alpha \\ & = \frac{1}{2}\int_{{{\kern 1pt} 0}}^{{{\kern 1pt} 1}} {\left[ {\left( {\left( {1 - \alpha } \right)a_{1} + \alpha a_{2} } \right)\left( {\left( {1 - \alpha } \right)b_{1} + \alpha b_{2} } \right) + \left( {\alpha a_{2} + \left( {1 - \alpha } \right)a_{3} } \right)\left( {\alpha b_{2} + \left( {1 - \alpha } \right)b_{3} } \right)} \right]} \text{d} \alpha \\ & = \frac{{2a_{1} b_{1} + a_{1} b_{2} + 3a_{2} b_{1} + 3a_{2} b_{3} + a_{3} b_{2} + 2a_{3} b_{3} }}{12}. \\ \end{aligned}$$

### **Proposition 4**

(Liu and Liu 2002) *Let*$\tilde{A}$*and*$\tilde{B}$*be two independent positive triangular fuzzy numbers, then*

9 $$E\left[ {m\tilde{A} + n\tilde{B}} \right] = mE\left[ {\tilde{A}} \right] + nE\left[ {\tilde{B}} \right].$$

*wheremandn are real numbers.*

## Notations and assumptions

In a single period setting, we examine a two-echelon supply chain in which a retailer orders the short life product from a manufacturer and sells it to customers in a high uncertain environment. The retailer has only one chance to order such product, since its selling season is much shorter than its order lead time. The uncertain demand faced by the retailer is assumed to be a positive triangular fuzzy number $\tilde{D} = \left( {d_{1} ,d_{2} ,d_{3} } \right)$ with $0 < d_{1} < d_{2} < d_{3}$. $d_{2}$ is the most possible value of demand $\tilde{D}$, this means that the demand is about *d*_2_. $d_{1}$ and $d_{3}$ are the minimum and maximum values of the demand, respectively. The fuzzy demand $\tilde{D}$ the following membership function $\mu_{{\tilde{D}}} (x)$:

10 $$\mu_{{\tilde{D}}} (x) = \left\{ {\begin{array}{*{20}l} {L(x),} \hfill &\quad {x \in \left[ {d_{1} ,d_{2} } \right],} \hfill \\ {R(x),} \hfill &\quad {x \in \left( {d_{2} ,d_{3} } \right],} \hfill \\ 0 \hfill &\quad {\text{otherwise} .} \hfill \\ \end{array} } \right.$$

For computational convenience, we use $L\left( x \right)$ and $R\left( x \right)$ denote the left and right spread functions of the fuzzy demand $\tilde{D}$. The values of $d_{1}$, $d_{2}$ and $d_{3}$ can be estimated by the decision makers.

The other notations used in this paper are as follows

$$q$$
Retailer’s order quantity;
$$\tilde{p}$$
Retailer’s fuzzy retail price of unit product;
$$\tilde{c}_{m}$$
Manufacturer’s fuzzy production cost of unit product;
$$\tilde{c}_{r}$$
Retailer’s fuzzy operational cost of unit product;
$$\tilde{v}$$
Unsold product’s fuzzy salvage value of unit product;
$$w$$
Manufacturer’s wholesale price of unit product;
$$\varPhi$$
Retailer’s percentage revenue sharing in the RS contract with $0 < \varPhi < 1$;
$$b$$
Manufacturer’s return price of unit unsold product in the return contract;
$$\tilde{\varPi }_{R}$$
Retailer’s fuzzy profit;
$$\tilde{\varPi }_{M}$$
Manufacturer’s fuzzy profit;
$$\tilde{\varPi }_{SC}$$
Supply chain system’s fuzzy profit

The assumptions related to this paper are given as follows:

### **Assumption 1**

The retailer and the manufacturer are both risk, they maximize the expected profits.

### **Assumption 2**

For any given $\alpha \in \left[ {0,1} \right]$, we assume $\tilde{p}_{R}^{ - 1} \left( \alpha \right) > \tilde{p}_{L}^{ - 1} \left( \alpha \right) > b > \tilde{v}_{R}^{ - 1} \left( \alpha \right) > \tilde{v}_{L}^{ - 1} \left( \alpha \right)$. These ensure that the retailer, the manufacturer and the supply chain system can obtain non-negative fuzzy profits.

## Models and solution approaches

In this section, we consider the centralized decision-making system and two types of supply chain contracts, namely, the revenue sharing contract and the return contract in a fuzzy decision environment, which can tell the manufacturer and the retailer how to set their optimal policies in this situation.

### Centralized decision-making system

We first consider the fuzzy centralized decision-making system. In this case, the manufacturer cooperates with the retailer and can be considered as the whole channel occupied by an integrated decision maker. Then, the profit for supply chain system is

11 $$\tilde{\varPi }_{SC} = \tilde{p}\hbox{min} \left( {q,\tilde{D}} \right) + \tilde{v}\hbox{max} \left( {q - \tilde{D},0} \right) - \tilde{c}_{m} q - \tilde{c}_{r} q.$$

where the profit $\tilde{\varPi }_{SC}$ is a fuzzy number since $\tilde{p}$, $\tilde{D}$, $\tilde{v}$, $\tilde{c}_{m}$ and $\tilde{c}_{r}$ are fuzzy numbers. Then, the manufacturer and the retailer choose their optimal order quantity $q$ to maximize the fuzzy expected profit $E\left[ {\tilde{\varPi }_{SC} } \right]$, which can be expressed as

$$\text{Max}_{q} E\left[ {\tilde{\varPi }_{SC} } \right] = E\left[ {\tilde{p}\,\hbox{min} \left( {q,\tilde{D}} \right) + \tilde{v}\,\hbox{max} \left( {q - \tilde{D},0} \right) - \tilde{c}_{m} q - \tilde{c}_{r} q} \right]$$

12 $$\text{s} .t.\quad {\kern 1pt} d_{1} \le q \le d_{3} .$$

Note that the fuzzy demand $\tilde{D} = (d_{1} ,d_{2} ,d_{3} )$ is a positive triangular fuzzy variable, then the optimal solution *q* set by the integrated decision maker has two conditions, $q \in \left[ {d_{1} ,d_{2} } \right]$ or $q \in \left( {d_{2} ,d_{3} } \right]$.

#### **Theorem 1**

*When*$q \in \left[ {d_{1} ,d_{2} } \right]$*, the optimal solution*$q^{*}$*satisfies the following equation*

13 $$\frac{1}{2}\int_{{{\kern 1pt} 0}}^{{{\kern 1pt} L\left( {q^{*} } \right)}} {\left( {\tilde{p}_{L}^{ - 1} \left( \alpha \right) - \tilde{v}_{R}^{ - 1} \left( \alpha \right)} \right)} \text{d} \alpha = E\left[ {\tilde{p}} \right] - E\left[ {\tilde{c}_{m} } \right] - E\left[ {\tilde{c}_{r} } \right].$$

#### *Proof*

If $q \in \left[ {d_{1} ,d_{2} } \right]$, we can get the $\alpha$ cut sets of $\hbox{min} \left( {q,\tilde{D}} \right)$ and $\hbox{max} \left( {q - \tilde{D},0} \right)$ as follows

$$\left( {\hbox{min} \left( {q,\tilde{D}} \right)} \right)\left( \alpha \right) = \left\{ \begin{aligned} \left[ {L^{ - 1} (\alpha ),\;q} \right],\;\;\;\;{\kern 1pt} \alpha \in \left[ {0,L\left( q \right)} \right], \hfill \\ \left[ {q,\;q} \right],\;\;\;{\kern 1pt} {\kern 1pt} {\kern 1pt} {\kern 1pt} {\kern 1pt} {\kern 1pt} {\kern 1pt} {\kern 1pt} {\kern 1pt} {\kern 1pt} {\kern 1pt} {\kern 1pt} {\kern 1pt} {\kern 1pt} {\kern 1pt} {\kern 1pt} {\kern 1pt} {\kern 1pt} {\kern 1pt} {\kern 1pt} {\kern 1pt} {\kern 1pt} {\kern 1pt} {\kern 1pt} {\kern 1pt} {\kern 1pt} {\kern 1pt} \alpha \in \left( {L\left( q \right),1} \right]. \hfill \\ \end{aligned} \right.$$

$$\left( {\hbox{max} \left( {q - \tilde{D},0} \right)} \right)\left( \alpha \right) = \left\{ \begin{aligned} \left[ {0,{\kern 1pt} q - L^{ - 1} (\alpha )} \right],\;\;\;\;{\kern 1pt} \alpha \in \left[ {0,L\left( q \right)} \right], \hfill \\ \left[ {0,\;0} \right],\;\;\;{\kern 1pt} {\kern 1pt} {\kern 1pt} {\kern 1pt} {\kern 1pt} {\kern 1pt} {\kern 1pt} {\kern 1pt} {\kern 1pt} {\kern 1pt} {\kern 1pt} {\kern 1pt} {\kern 1pt} {\kern 1pt} {\kern 1pt} {\kern 1pt} {\kern 1pt} {\kern 1pt} {\kern 1pt} {\kern 1pt} {\kern 1pt} {\kern 1pt} {\kern 1pt} {\kern 1pt} {\kern 1pt} {\kern 1pt} {\kern 1pt} {\kern 1pt} {\kern 1pt} {\kern 1pt} {\kern 1pt} {\kern 1pt} {\kern 1pt} {\kern 1pt} {\kern 1pt} {\kern 1pt} {\kern 1pt} {\kern 1pt} {\kern 1pt} {\kern 1pt} \alpha \in \left( {L\left( q \right),1} \right]. \hfill \\ \end{aligned} \right.$$

If $\alpha \in \left[ {0,L\left( q \right)} \right]$, then the $\alpha$ cut set of $\tilde{\varPi }_{SC} \left( \alpha \right)$ can be obtained as

$$\begin{aligned} \tilde{\varPi}_{SC} \left(\alpha \right) & = \left[{\tilde{p}_{L}^{- 1} \left(\alpha \right)L^{- 1} \left(\alpha \right),\tilde{p}_{R}^{- 1} \left(\alpha \right)q} \right] + \left[{0,\tilde{v}_{R}^{- 1} \left(\alpha \right)q - \tilde{v}_{R}^{- 1} \left(\alpha \right)L^{- 1} \left(\alpha \right)} \right] \\ & \quad - \left[{\left({\tilde{c}_{m}} \right)_{L}^{- 1} \left(\alpha \right)q,\left({\tilde{c}_{m}} \right)_{R}^{- 1} \left(\alpha \right)q} \right] - \left[{\left({\tilde{c}_{r}} \right)_{L}^{- 1} \left(\alpha \right)q,\left({\tilde{c}_{r}} \right)_{R}^{- 1} \left(\alpha \right)q} \right] \\ & = \left[{\tilde{p}_{L}^{- 1} \left(\alpha \right)L^{- 1} \left(\alpha \right) - \left({\tilde{c}_{m}} \right)_{R}^{- 1} \left(\alpha \right)q - \left({\tilde{c}_{r}} \right)_{R}^{- 1} \left(\alpha \right)q} \right., \\ & \left.\quad\quad \tilde{p}_{R}^{- 1} \left(\alpha \right)q + \tilde{v}_{R}^{- 1} \left(\alpha \right)q - \tilde{v}_{R}^{- 1} \left(\alpha \right)L^{- 1} \left(\alpha \right) - \left({\tilde{c}_{m}} \right)_{L}^{- 1} \left(\alpha \right)q - \left({\tilde{c}_{r}} \right)_{L}^{- 1} \left(\alpha \right)q \right]. \\ \end{aligned}$$

If ${\kern 1pt} \alpha \in \left( {L\left( q \right),1} \right]$, then the $\alpha$ cut set of $\tilde{\varPi }_{SC} \left( \alpha \right)$ can be derived as

$$\begin{aligned} \tilde{\varPi }_{SC} \left( \alpha \right) & = \left[ {\tilde{p}_{L}^{ - 1} \left( \alpha \right)q,\tilde{p}_{R}^{ - 1} \left( \alpha \right)q} \right] - \left[ {\left( {\tilde{c}_{m} } \right)_{L}^{ - 1} \left( \alpha \right)q,\left( {\tilde{c}_{m} } \right)_{R}^{ - 1} \left( \alpha \right)q} \right] - \left[ {\left( {\tilde{c}_{r} } \right)_{L}^{ - 1} \left( \alpha \right)q,\left( {\tilde{c}_{r} } \right)_{R}^{ - 1} \left( \alpha \right)q} \right] \\ & = \left[ {\tilde{p}_{L}^{ - 1} \left( \alpha \right)q - \left( {\tilde{c}_{m} } \right)_{R}^{ - 1} \left( \alpha \right)q - \left( {\tilde{c}_{r} } \right)_{R}^{ - 1} \left( \alpha \right)q,{\kern 1pt} {\kern 1pt} {\kern 1pt} {\kern 1pt} {\kern 1pt} \tilde{p}_{R}^{ - 1} \left( \alpha \right)q - \left( {\tilde{c}_{m} } \right)_{L}^{ - 1} \left( \alpha \right)q - \left( {\tilde{c}_{r} } \right)_{L}^{ - 1} \left( \alpha \right)q} \right]. \\ \end{aligned}$$

According to Proposition 3, the fuzzy expected profit $E\left[ {\tilde{\varPi }_{SC} } \right]$ is

14 $$\begin{aligned} E\left[{\tilde{\varPi}_{SC}} \right] & = \frac{1}{2}\int_{{{\kern 1pt} 0}}^{{{\kern 1pt} L\left(q \right)}} {\left({\tilde{p}_{L}^{- 1} \left(\alpha \right)L^{- 1} \left(\alpha \right) - \left({\tilde{c}_{m}} \right)_{R}^{- 1} \left(\alpha \right)q - \left({\tilde{c}_{r}} \right)_{R}^{- 1} \left(\alpha \right)q} \right.} + \tilde{p}_{R}^{- 1} \left(\alpha \right)q \\ &\left. \quad +\, \tilde{v}_{R}^{- 1} \left(\alpha \right)q - \tilde{v}_{R}^{- 1} \left(\alpha \right)L^{- 1} \left(\alpha \right) - \left({\tilde{c}_{m}} \right)_{L}^{- 1} \left(\alpha \right)q - \left({\tilde{c}_{r}} \right)_{L}^{- 1} \left(\alpha \right)q \right)\text{d} \alpha \\ & \quad +\, \frac{1}{2}\int_{{{\kern 1pt} L\left(q \right)}}^{{{\kern 1pt} 1}} {\left({\tilde{p}_{L}^{- 1} \left(\alpha \right)q - \left({\tilde{c}_{m}} \right)_{R}^{- 1} \left(\alpha \right)q - \left({\tilde{c}_{r}} \right)_{R}^{- 1} \left(\alpha \right)q + {\kern 1pt} {\kern 1pt} \tilde{p}_{R}^{- 1} \left(\alpha \right)q - \left({\tilde{c}_{m}} \right)_{L}^{- 1} \left(\alpha \right)q - \left({\tilde{c}_{r}} \right)_{L}^{- 1} \left(\alpha \right)q} \right)} \text{d} \alpha \\ & = - \frac{1}{2}\int_{{{\kern 1pt} 0}}^{{{\kern 1pt} L\left(q \right)}} {\left({\tilde{p}_{L}^{- 1} \left(\alpha \right) - \tilde{v}_{R}^{- 1} \left(\alpha \right)} \right)} \left({q - L^{- 1} \left(\alpha \right)} \right)\text{d} \alpha + \left({E\left[{\tilde{p}} \right] - E\left[{\tilde{c}_{m}} \right] - E\left[{\tilde{c}_{r}} \right]} \right)q. \\ \end{aligned}$$

The first-order and second-order derivatives $E\left[ {\tilde{\varPi }_{SC} } \right]$ about $q$ are

$$\begin{aligned} & \frac{{\text{d} E\left[ {\tilde{\varPi }_{SC} } \right]}}{{\text{d} q}} = - \frac{1}{2}\int_{{{\kern 1pt} 0}}^{{{\kern 1pt} L\left( q \right)}} {\left( {\tilde{p}_{L}^{ - 1} \left( \alpha \right) - \tilde{v}_{R}^{ - 1} \left( \alpha \right)} \right)} \text{d} \alpha + E\left[ {\tilde{p}} \right] - E\left[ {\tilde{c}_{m} } \right] - E\left[ {\tilde{c}_{r} } \right], \\ & \frac{{\text{d}^{2} E\left[ {\tilde{\varPi }_{SC} } \right]}}{{\text{d} q^{2} }} = - \frac{1}{2}\left( {\tilde{p}_{L}^{ - 1} \left( {L\left( q \right)} \right) - \tilde{v}_{R}^{ - 1} \left( {L\left( q \right)} \right)} \right)L^{'} \left( q \right). \\ \end{aligned}$$

Since $L\left( q \right)$ is an increasing function about $q$ with $L^{{\prime }} \left( q \right) > 0$, therefore $\frac{{\text{d}^{2} E\left[ {\tilde{\varPi }_{SC} } \right]}}{{\text{d} q^{2} }} < 0$ and $E\left[ {\tilde{\varPi }_{SC} } \right]$ is a concave function with respect to $q$ for $q \in \left[ {d_{1} ,d_{2} } \right]$.

Hence, let $\frac{{\text{d} E\left[ {\tilde{\varPi }_{SC} } \right]}}{{\text{d} q}} = 0$, we can derive the optimal order quantity $q^{*}$ as

15 $$- \frac{1}{2}\int_{{{\kern 1pt} 0}}^{{{\kern 1pt} L\left( {q^{*} } \right)}} {\left( {\tilde{p}_{L}^{ - 1} \left( \alpha \right) - \tilde{v}_{R}^{ - 1} \left( \alpha \right)} \right)} \text{d} \alpha + E\left[ {\tilde{p}} \right] - E\left[ {\tilde{c}_{m} } \right] - E\left[ {\tilde{c}_{r} } \right] = 0.$$

Solving Eq. (15), the optimal order quantity $q^{*}$ can be derived as shown in Eq. (13).

Theorem 1 is proved.

#### **Theorem 2**

*When*$q \in \left( {d_{2} ,d_{3} } \right]$*, the optimal solution*$q^{*}$*satisfies the following equation*

16 $$\frac{1}{2}\int_{{{\kern 1pt} 0}}^{{{\kern 1pt} R\left( {q^{*} } \right)}} {\left( {\tilde{p}_{L}^{ - 1} \left( \alpha \right) - \tilde{v}_{R}^{ - 1} \left( \alpha \right)} \right)} \text{d} \alpha = E\left[ {\tilde{c}_{m} } \right] + E\left[ {\tilde{c}_{r} } \right] - E\left[ {\tilde{v}} \right].$$

#### *Proof*

If $q \in \left( {d_{2} ,d_{3} } \right]$, we can get the $\alpha$ cut sets of $\hbox{min} \left( {q,\tilde{D}} \right)$ and $\hbox{max} \left( {q - \tilde{D},0} \right)$ as follows

$$\left( {\hbox{min} \left( {q,\tilde{D}} \right)} \right)\left( \alpha \right) = \left\{ \begin{aligned} \left[ {L^{ - 1} (\alpha ),\;q} \right],\;\;\;\;{\kern 1pt} {\kern 1pt} {\kern 1pt} {\kern 1pt} {\kern 1pt} {\kern 1pt} {\kern 1pt} {\kern 1pt} {\kern 1pt} {\kern 1pt} {\kern 1pt} {\kern 1pt} {\kern 1pt} {\kern 1pt} {\kern 1pt} {\kern 1pt} {\kern 1pt} {\kern 1pt} {\kern 1pt} {\kern 1pt} {\kern 1pt} \alpha \in \left[ {0,R\left( q \right)} \right], \hfill \\ \left[ {L^{ - 1} (\alpha ),\;R^{ - 1} (\alpha )} \right],\;{\kern 1pt} {\kern 1pt} {\kern 1pt} {\kern 1pt} {\kern 1pt} {\kern 1pt} {\kern 1pt} {\kern 1pt} \alpha \in \left( {R\left( q \right),1} \right]. \hfill \\ \end{aligned} \right.$$

$$\left( {\hbox{max} \left( {q - \tilde{D},0} \right)} \right)\left( \alpha \right) = \left\{ \begin{aligned} \left[ {0,\;q - L^{ - 1} \left( \alpha \right)} \right],\;\;\;\;{\kern 1pt} {\kern 1pt} {\kern 1pt} {\kern 1pt} {\kern 1pt} {\kern 1pt} {\kern 1pt} {\kern 1pt} {\kern 1pt} {\kern 1pt} {\kern 1pt} {\kern 1pt} {\kern 1pt} {\kern 1pt} {\kern 1pt} {\kern 1pt} {\kern 1pt} {\kern 1pt} {\kern 1pt} {\kern 1pt} {\kern 1pt} {\kern 1pt} {\kern 1pt} {\kern 1pt} {\kern 1pt} {\kern 1pt} {\kern 1pt} {\kern 1pt} {\kern 1pt} {\kern 1pt} {\kern 1pt} {\kern 1pt} {\kern 1pt} {\kern 1pt} {\kern 1pt} \alpha \in \left[ {0,R\left( q \right)} \right], \hfill \\ \left[ {q - R^{ - 1} \left( \alpha \right),\;q - L^{ - 1} \left( \alpha \right)} \right],\;\;{\kern 1pt} {\kern 1pt} {\kern 1pt} \alpha \in \left( {R\left( q \right),1} \right]. \hfill \\ \end{aligned} \right.$$

If $\alpha \in \left[ {0,R\left( q \right)} \right]$, then the $\alpha$ cut set of the fuzzy profit $\tilde{\varPi }_{SC} \left( \alpha \right)$ can be obtained as

$$\begin{aligned} \tilde{\varPi}_{SC} \left(\alpha \right) & = \left[{\tilde{p}_{L}^{- 1} \left(\alpha \right)L^{- 1} \left(\alpha \right),\tilde{p}_{R}^{- 1} \left(\alpha \right)q} \right] + \left[{0,\tilde{v}_{R}^{- 1} \left(\alpha \right)q - \tilde{v}_{R}^{- 1} \left(\alpha \right)L^{- 1} \left(\alpha \right)} \right] \\ & \quad - \left[{\left({\tilde{c}_{m}} \right)_{L}^{- 1} \left(\alpha \right)q,\left({\tilde{c}_{m}} \right)_{R}^{- 1} \left(\alpha \right)q} \right] - \left[{\left({\tilde{c}_{r}} \right)_{L}^{- 1} \left(\alpha \right)q,\left({\tilde{c}_{r}} \right)_{R}^{- 1} \left(\alpha \right)q} \right] \\ & = \left[{\tilde{p}_{L}^{- 1} \left(\alpha \right)L^{- 1} \left(\alpha \right) -} \right.\left({\tilde{c}_{m}} \right)_{R}^{- 1} \left(\alpha \right)q - \left({\tilde{c}_{r}} \right)_{R}^{- 1} \left(\alpha \right)q, \\ & \left. \quad\quad \tilde{p}_{R}^{- 1} \left(\alpha \right)q + \tilde{v}_{R}^{- 1} \left(\alpha \right)q - \tilde{v}_{R}^{- 1} \left(\alpha \right)L^{- 1} \left(\alpha \right) - \left({\tilde{c}_{m}} \right)_{L}^{- 1} \left(\alpha \right)q - \left({\tilde{c}_{r}} \right)_{L}^{- 1} \left(\alpha \right)q \right]. \\ \end{aligned}$$

If ${\kern 1pt} \alpha \in \left( {R\left( q \right),1} \right]$, then the $\alpha$ cut set of the fuzzy profit $\tilde{\varPi }_{SC} \left( \alpha \right)$ can be derived as

$$\begin{aligned} \tilde{\varPi}_{SC} \left(\alpha \right) & = \left[{\tilde{p}_{L}^{- 1} \left(\alpha \right)L^{- 1} \left(\alpha \right),\tilde{p}_{R}^{- 1} \left(\alpha \right)R^{- 1} \left(\alpha \right)} \right] + \left[{\tilde{v}_{L}^{- 1} \left(\alpha \right)q - \tilde{v}_{L}^{- 1} \left(\alpha \right)R^{- 1} \left(\alpha \right),\tilde{v}_{R}^{- 1} \left(\alpha \right)q - \tilde{v}_{R}^{- 1} \left(\alpha \right)L^{- 1} \left(\alpha \right)} \right] \\ & \quad - \left[{\left({\tilde{c}_{m}} \right)_{L}^{- 1} \left(\alpha \right)q,\left({\tilde{c}_{m}} \right)_{R}^{- 1} \left(\alpha \right)q} \right] - \left[{\left({\tilde{c}_{r}} \right)_{L}^{- 1} \left(\alpha \right)q,\left({\tilde{c}_{r}} \right)_{R}^{- 1} \left(\alpha \right)q} \right] \\ & = \left[{\tilde{p}_{L}^{- 1} \left(\alpha \right)L^{- 1} \left(\alpha \right) + \tilde{v}_{L}^{- 1} \left(\alpha \right)q - \tilde{v}_{L}^{- 1} \left(\alpha \right)R^{- 1} \left(\alpha \right) -} \right.\left({\tilde{c}_{m}} \right)_{R}^{- 1} \left(\alpha \right)q - \left({\tilde{c}_{r}} \right)_{R}^{- 1} \left(\alpha \right)q, \\ & \left.\quad\quad \tilde{p}_{R}^{- 1} \left(\alpha \right)R^{- 1} \left(\alpha \right) + \tilde{v}_{R}^{- 1} \left(\alpha \right)q - \tilde{v}_{R}^{- 1} \left(\alpha \right)L^{- 1} \left(\alpha \right) - \left({\tilde{c}_{m}} \right)_{L}^{- 1} \left(\alpha \right)q - \left({\tilde{c}_{r}} \right)_{L}^{- 1} \left(\alpha \right)q \right]. \\ \end{aligned}$$

According to Proposition 3, the fuzzy expected profit $E\left[ {\tilde{\varPi }_{SC} } \right]$ is

17 $$\begin{aligned} E\left[{\tilde{\varPi}_{SC}} \right] & =\frac{1}{2}\int_{{{\kern 1pt} 0}}^{{{\kern 1pt} R\left(q \right)}}{\left({\tilde{p}_{L}^{- 1} \left(\alpha \right)L^{- 1}\left(\alpha \right) -} \right.} \left({\tilde{c}_{m}}\right)_{R}^{- 1} \left(\alpha \right)q - \left({\tilde{c}_{r}} \right)_{R}^{- 1} \left(\alpha \right)q + \tilde{p}_{R}^{-1} \left(\alpha \right)q \\ & \left.\quad +\, \tilde{v}_{R}^{- 1} \left(\alpha \right)q - \tilde{v}_{R}^{- 1} \left(\alpha\right)L^{- 1} \left(\alpha \right) - \left({\tilde{c}_{m}}\right)_{L}^{- 1} \left(\alpha \right)q - \left({\tilde{c}_{r}} \right)_{L}^{- 1} \left(\alpha \right)q \right)\text{d} \alpha\\ & \quad +\, \frac{1}{2}\int_{{{\kern 1pt} R\left(q\right)}}^{{{\kern 1pt} 1}} {\left({\tilde{p}_{L}^{- 1} \left(\alpha \right)L^{- 1} \left(\alpha \right) + \tilde{v}_{L}^{- 1} \left(\alpha \right)q -} \right.} \tilde{v}_{L}^{- 1}\left(\alpha \right)R^{- 1} \left(\alpha \right) - \left({\tilde{c}_{m}} \right)_{R}^{- 1} \left(\alpha \right)q -\left({\tilde{c}_{r}} \right)_{R}^{- 1} \left(\alpha \right)q\\ &\left. \times\quad \tilde{p}_{R}^{- 1} \left(\alpha\right)R^{- 1} \left(\alpha \right) + \tilde{v}_{R}^{- 1}\left(\alpha \right)q - \tilde{v}_{R}^{- 1} \left(\alpha\right)L^{- 1} \left(\alpha \right) - \left({\tilde{c}_{m}}\right)_{L}^{- 1} \left(\alpha \right)q - \left({\tilde{c}_{r}} \right)_{L}^{- 1} \left(\alpha \right)q \right)\text{d} \alpha\\ & = \frac{1}{2}\int_{{{\kern 1pt} 0}}^{{{\kern 1pt} R\left(q \right)}} {\left({\tilde{p}_{R}^{- 1} \left(\alpha \right) -\tilde{v}_{l}^{- 1} \left(\alpha \right)} \right)\left({q -R^{- 1} \left(\alpha \right)} \right)} \text{d} \alpha +\left({E\left[{\tilde{v}} \right] - E\left[{\tilde{c}_{m}}\right] - E\left[{\tilde{c}_{r}} \right]} \right)q \\ & \quad+ E\left[{\tilde{p}\tilde{D}} \right] - \frac{1}{2}\int_{{{\kern 1pt} 0}}^{{{\kern 1pt} 1}} {\left({\tilde{v}_{L}^{- 1} \left(\alpha \right)R^{- 1} \left(\alpha \right) + \tilde{v}_{R}^{- 1} \left(\alpha \right)L^{- 1} \left(\alpha \right)} \right)} \text{d} \alpha. \\\end{aligned}$$

The first-order and second-order derivatives $E\left[ {\tilde{\varPi }_{SC} } \right]$ about $q$ are

$$\begin{aligned} & \frac{{\text{d} E\left[ {\tilde{\varPi }_{SC} } \right]}}{{\text{d} q}} = \frac{1}{2}\int_{{{\kern 1pt} 0}}^{{{\kern 1pt} R\left( q \right)}} {\left( {\tilde{p}_{R}^{ - 1} \left( \alpha \right) - \tilde{v}_{L}^{ - 1} \left( \alpha \right)} \right)} \text{d} \alpha + E\left[ {\tilde{v}} \right] - E\left[ {\tilde{c}_{m} } \right] - E\left[ {\tilde{c}_{r} } \right], \\ & \frac{{\text{d}^{2} E\left[ {\tilde{\varPi }_{SC} } \right]}}{{\text{d} q^{2} }} = \frac{1}{2}\left( {\tilde{p}_{R}^{ - 1} \left( {R\left( q \right)} \right) - \tilde{v}_{L}^{ - 1} \left( {R\left( q \right)} \right)} \right)R^{{\prime }} \left( q \right). \\ \end{aligned}$$

Since $R\left( q \right)$ is a decreasing function about $q$ with $R^{{\prime }} \left( q \right) < 0$, therefore $\frac{{\text{d}^{2} E\left[ {\tilde{\varPi }_{SC} } \right]}}{{\text{d} q^{2} }} < 0$ and $E\left[ {\tilde{\varPi }_{SC} } \right]$ is a concave function with respect to $q$ for $q \in \left( {d_{2} ,d_{3} } \right]$.

Hence, let $\frac{{\text{d} E\left[ {\tilde{\varPi }_{SC} } \right]}}{{\text{d} q}} = 0$, we can derive the optimal order quantity $q^{*}$ as

18 $$\frac{1}{2}\int_{{{\kern 1pt} 0}}^{{{\kern 1pt} R\left( q \right)}} {\left( {\tilde{p}_{R}^{ - 1} \left( \alpha \right) - \tilde{v}_{L}^{ - 1} \left( \alpha \right)} \right)} \text{d} \alpha + E\left[ {\tilde{v}} \right] - E\left[ {\tilde{c}_{m} } \right] - E\left[ {\tilde{c}_{r} } \right] = 0.$$

Solving Eq. (18), the optimal order quantity $q^{*}$ can be obtained as shown in Eq. (16).

Theorem 2 is proved.

Here, we further consider the fuzzy parameters $\tilde{p}$, $\tilde{v}$, $\tilde{c}_{m}$ and $\tilde{c}_{r}$ as triangular fuzzy numbers as follows

$$\tilde{p} = \left( {p - \Delta_{11} ,p,p + \Delta_{12} } \right),\quad \tilde{v} = \left( {v - \Delta_{21} ,v,v + \Delta_{22} } \right),\quad \tilde{c}_{m} = \left( {c_{m} - \Delta_{31} ,c_{m} ,c_{m} + \Delta_{32} } \right),\quad {\text{and}}\quad \tilde{c}_{r} = \left( {c_{r} - \Delta_{41} ,c_{r} ,c_{r} + \Delta_{42} } \right).$$

where $$\Delta_{ij} > 0$$, $$i = 1,2,3,4$$, $$j = 1,2$$.

According to Eqs. (3) and (7), the $\alpha$ cut sets of the fuzzy parameters $\tilde{p}$, $\tilde{v}$, $\tilde{c}_{m}$, $\tilde{c}_{r}$, and their fuzzy expected values can be obtained as

$$\tilde{p}_{L}^{ - 1} \left( \alpha \right) = p - \Delta_{11} + \Delta_{11} \alpha ,\quad \tilde{p}_{R}^{ - 1} \left( \alpha \right) = p + \Delta_{12} - \Delta_{12} \alpha ,\quad {\text{and}}\quad E\left[ {\tilde{p}} \right] = p + 0.25\Delta_{12} - 0.25\Delta_{11} .$$

$$\tilde{v}_{L}^{ - 1} \left( \alpha \right) = v - \Delta_{21} + \Delta_{21} \alpha ,\quad \tilde{v}_{R}^{ - 1} \left( \alpha \right) = v + \Delta_{22} - \Delta_{22} \alpha ,\quad {\text{and}}\quad E\left[ {\tilde{v}} \right] = v + 0.25\Delta_{22} - 0.25\Delta_{21} .$$

$$\left( {\tilde{c}_{m} } \right)_{L}^{ - 1} \left( \alpha \right) = c_{m} - \Delta_{31} + \Delta_{31} \alpha ,\quad \left( {\tilde{c}_{m} } \right)_{R}^{ - 1} \left( \alpha \right) = c_{m} + \Delta_{32} - \Delta_{32} \alpha ,\quad {\text{and}}\quad E\left[ {\tilde{c}_{m} } \right] = c_{m} + 0.25\Delta_{32} - 0.25\Delta_{31} .$$

$$\left( {\tilde{c}_{r} } \right)_{L}^{ - 1} \left( \alpha \right) = c_{r} - \Delta_{41} + \Delta_{41} \alpha ,\quad \left( {\tilde{c}_{r} } \right)_{R}^{ - 1} \left( \alpha \right) = c_{r} + \Delta_{42} - \Delta_{42} \alpha ,\quad {\text{and}}\quad E\left[ {\tilde{c}_{r} } \right] = c_{r} + 0.25\Delta_{42} - 0.25\Delta_{41} .$$

Then, the Theorems 1 and 2 can be translated into the following Theorems 3 and 4, respectively.

#### **Theorem 3**

*When*$c_{m} + c_{r} + \Delta_{1} < p \le 2\left( {c_{m} + c_{r} } \right) - v - 2\Delta_{2}$*, the optimal order quantity*$q^{*}$*can be expressed as*

19 $$q^{*} = L^{ - 1} \left( {\frac{{ - 0.5\left( {p - v - \Delta_{11} - \Delta_{22} } \right) + \sqrt {0.25\left( {p - v - \Delta_{11} - \Delta_{22} } \right)^{2} + \left( {\Delta_{11} + \Delta_{22} } \right)\left( {p - c_{m} - c_{r} - \Delta_{1} } \right)} }}{{0.5\left( {\Delta_{11} + \Delta_{22} } \right)}}} \right).$$

*where*

$$\begin{aligned} & \Delta_{1} = 0.25\Delta_{11} - 0.25\Delta_{12} - 0.25\Delta_{31} + 0.25\Delta_{32} - 0.25\Delta_{41} + 0.25\Delta_{2} , \\ & \Delta_{2} = 0.25\Delta_{12} + 0.25\Delta_{22} + 0.25\Delta_{31} - 0.25\Delta_{32} + 0.25\Delta_{41} - 0.25\Delta_{42} , \\ \end{aligned}$$

$L^{ - 1}$*is the inverse function of*$L$.

#### *Proof*

When $q \in \left[ {d_{1} ,d_{2} } \right]$, substituting $\tilde{p}_{L}^{ - 1} (\alpha )$, $\tilde{v}_{R}^{ - 1} (\alpha )$, $E\left[ {\tilde{p}} \right]$, $E\left[ {\tilde{c}_{m} } \right]$ and $E\left[ {\tilde{c}_{r} } \right]$ into Eq. (13), we can obtain

20 $$0.25\left( {\Delta_{11} + \Delta_{22} } \right)L^{2} \left( {q^{*} } \right) + 0.5\left( {p - v - \Delta_{11} - \Delta_{22} } \right)L\left( {q^{*} } \right) - \left( {p - c_{m} - c_{r} - \Delta_{1} } \right) = 0.$$

where

$$\Delta_{1} = 0.25\Delta_{11} - 0.25\Delta_{12} - 0.25\Delta_{31} + 0.25\Delta_{32} - 0.25\Delta_{41} + 0.25\Delta_{42} .$$

Solving Eq. (20) leads to

$$L\left( {q^{*} } \right) = \frac{{ - 0.5\left( {p - v - \Delta_{11} - \Delta_{22} } \right) + \sqrt {0.25\left( {p - v - \Delta_{11} - \Delta_{22} } \right)^{2} + \left( {\Delta_{11} + \Delta_{22} } \right)\left( {p - c_{m} - c_{r} - \Delta_{1} } \right)} }}{{0.5\left( {\Delta_{11} + \Delta_{22} } \right)}}.$$

Due to $0 \le L\left( {q^{*} } \right) \le 1$, we have $p \le 2\left( {c_{m} + c_{r} } \right) - v - 2\Delta_{2} .$ where

$$\Delta_{2} = 0.25\Delta_{12} + 0.25\Delta_{22} + 0.25\Delta_{31} - 0.25\Delta_{32} + 0.25\Delta_{41} - 0.25\Delta_{42} .$$

In addition, in order to let the supply chain members obtain the positive fuzzy profits, $E\left[ {\tilde{p}} \right] > E\left[ {\tilde{c}_{m} } \right] + E\left[ {\tilde{c}_{r} } \right]$ must hold.

That is $$p > c_{m} + c_{r} + \Delta_{1} .$$

Theorem 3 is proved.

#### **Theorem 4**

*When*$p \ge 2\left( {c_{m} + c_{r} } \right) - v - 2\Delta_{2}$*, the optimal order quantity*$q^{*}$*can be expressed as*

21 $$q^{*} = R^{ - 1} \left( {\frac{{0.5\left( {p - v + \Delta_{12} + \Delta_{21} } \right) - \sqrt {0.25\left( {p - v + \Delta_{12} + \Delta_{21} } \right)^{2} - \left( {\Delta_{12} + \Delta_{21} } \right)\left( {c_{m} + c_{r} - v + \Delta_{3} } \right)} }}{{0.5\left( {\Delta_{12} + \Delta_{21} } \right)}}} \right).$$

*where*

$$\Delta_{3} = 0.25\Delta_{21} - 0.25\Delta_{22} - 0.25\Delta_{31} + 0.25\Delta_{32} - 0.25\Delta_{41} + 0.25\Delta_{42} ,$$

$R^{ - 1}$*is the inverse function of*$R$.

#### *Proof*

When $q \in \left( {d_{2} ,d_{3} } \right]$, Substituting $\tilde{p}_{R}^{ - 1} (\alpha )$, $\tilde{v}_{L}^{ - 1} (\alpha )$, $E\left[ {\tilde{c}_{m} } \right]$, $E\left[ {\tilde{c}_{r} } \right]$ and $E\left[ {\tilde{v}} \right]$ into Eq. (13), we can obtain

22 $$- 0.25\left( {\Delta_{12} + \Delta_{21} } \right)R^{2} \left( {q^{*} } \right) + 0.5\left( {p - v + \Delta_{12} + \Delta_{21} } \right)R\left( {q^{*} } \right) - \left( {c_{m} + c_{r} - v + \Delta_{3} } \right) = 0.$$

where

$$\Delta_{3} = 0.25\Delta_{21} - 0.25\Delta_{22} - 0.25\Delta_{31} + 0.25\Delta_{32} - 0.25\Delta_{41} + 0.25\Delta_{42} .$$

Solving Eq. (22) leads to

$$R\left( {q^{*} } \right) = \frac{{0.5\left( {p - v + \Delta_{12} + \Delta_{21} } \right) - \sqrt {0.25\left( {p - v + \Delta_{12} + \Delta_{21} } \right)^{2} - \left( {\Delta_{12} + \Delta_{21} } \right)\left( {c_{m} + c_{r} - v + \Delta_{3} } \right)} }}{{0.5\left( {\Delta_{12} + \Delta_{21} } \right)}}.$$

Due to $0 \le R\left( {q^{*} } \right) \le 1$, we have $p \ge 2\left( {c_{m} + c_{r} } \right) - v - 2\Delta_{2} .$

Theorem 4 is proved.

#### **Proposition 5**

*If*$p = 2\left( {c_{m} + c_{r} } \right) - v - 2\Delta_{2}$*, then*$L\left( {q^{*} } \right) = R\left( {q^{*} } \right).$ Proposition 5 *reveals that the optimal order quantity*$q^{*}$*is a continuous function with respect to the retail price.*

#### **Proposition 6**

*If*$\Delta_{ij} \to 0$, $i = 1,2,3,4$, $j = 1,2$, *then the fuzzy parameters*$\tilde{p}$, $\tilde{v}$, $\tilde{c}_{m}$, $\tilde{c}_{r}$*degenerate to crisp values, and the results in Theorems 3 and 4 degenerate to*

$$q^{*} = \left\{ {\begin{array}{*{20}l} {L^{ - 1} \left( {\frac{{2\left( {p - c_{m} - c_{r} } \right)}}{p - v}} \right),} \hfill & \quad{p \in \left( {c_{m} + c_{r} ,2\left( {c_{m} + c_{r} } \right) - v} \right],} \hfill \\ {R^{ - 1} \left( {\frac{{2\left( {c_{m} + c_{r} - v} \right)}}{p - v}} \right),} \hfill &\quad {p \in \left[ {2\left( {c_{m} + c_{r} } \right) - v, + \infty } \right).} \hfill \\ \end{array} } \right.$$

*There are just the solutions in a fuzzy demand environment.*

#### *Proof*

Case 1 $$q \in \left[ {d_{1} ,d_{2} } \right]$$

If $q \in \left[ {d_{1} ,d_{2} } \right]$, that is $c_{m} + c_{r} + \Delta_{1} < p \le 2\left( {c_{m} + c_{r} } \right) - v - 2\Delta_{2}$, then let $\Delta_{ij} \to 0$, we can have

$$\begin{aligned} q^{*} & = \mathop {\lim }\limits_{{\Delta_{ij} \to 0}} L^{ - 1} \left( {\frac{{ - 0.5\left( {p - v - \Delta_{11} - \Delta_{22} } \right) + \sqrt {0.25\left( {p - v - \Delta_{11} - \Delta_{22} } \right)^{2} + \left( {\Delta_{11} + \Delta_{22} } \right)\left( {p - c_{m} - c_{r} - \Delta_{1} } \right)} }}{{0.5\left( {\Delta_{11} + \Delta_{22} } \right)}}} \right) \\ & = \mathop {\lim }\limits_{{\Delta_{ij} \to 0}} L^{ - 1} \left( {\frac{{0.5 + \tfrac{{ - 0.5\left( {p - v} \right) + \left( {p - c_{m} - c_{r} } \right)}}{{2\sqrt {0.25\left( {p - v - \Delta_{11} - \Delta_{22} } \right)^{2} + \left( {\Delta_{11} + \Delta_{22} } \right)\left( {p - c_{m} - c_{r} - \Delta_{1} } \right)} }}}}{0.5}} \right) \\ & = L^{ - 1} \left( {\frac{{0.5 + \tfrac{{ - 0.5\left( {p - v} \right) + \left( {p - c_{m} - c_{r} } \right)}}{p - v}}}{0.5}} \right) \\ & = L^{ - 1} \left( {\frac{{2\left( {p - c_{m} - c_{r} } \right)}}{p - v}} \right). \\ \end{aligned}$$

Case 2. $$q \in \left( {d_{2} ,d_{3} } \right]$$

If $q \in \left( {d_{2} ,d_{3} } \right]$, that is $p \ge 2\left( {c_{m} + c_{r} } \right) - v - 2\Delta_{2}$, then let $\Delta_{ij} \to 0$, we can have

$$\begin{aligned} q^{*} & = \mathop {\lim }\limits_{{\Delta_{ij} \to 0}} R^{ - 1} \left( {\frac{{0.5\left( {p - v + \Delta_{12} + \Delta_{21} } \right) - \sqrt {0.25\left( {p - v + \Delta_{12} + \Delta_{21} } \right)^{2} - \left( {\Delta_{12} + \Delta_{21} } \right)\left( {c_{m} + c_{r} - v + \Delta_{3} } \right)} }}{{0.5\left( {\Delta_{12} + \Delta_{21} } \right)}}} \right) \\ & = \mathop {\lim }\limits_{{\Delta_{ij} \to 0}} R^{ - 1} \left( {\frac{{0.5 - \tfrac{{0.5\left( {p - v} \right) - \left( {c_{m} + c_{r} - v} \right)}}{{2\sqrt {0.25\left( {p - v + \Delta_{12} + \Delta_{21} } \right)^{2} - \left( {\Delta_{12} + \Delta_{21} } \right)\left( {c_{m} + c_{r} - v + \Delta_{3} } \right)} }}}}{0.5}} \right) \\ & = R^{ - 1} \left( {\frac{{0.5 - \tfrac{{0.5\left( {p - v} \right) - \left( {c_{m} + c_{r} - v} \right)}}{p - v}}}{0.5}} \right) \\ & = R^{ - 1} \left( {\frac{{2\left( {c_{m} + c_{r} - v} \right)}}{p - v}} \right). \\ \end{aligned}$$

Proposition 6 is proved.

The result of Proposition 6 is similar to that of the works by Xu and Zhai (2010), Ye and Li (2011). They only consider the demand as a fuzzy number. Compared to their models, the parameters $\tilde{D}$, $\tilde{p}$, $\tilde{v}$, $\tilde{c}_{m}$ and $\tilde{c}_{r}$ are all assumed to be the triangular fuzzy numbers in our model, and their models are special cases of this paper.

From Eqs. (13), (14), (16) and (17), the optimal fuzzy expected profit $E\left[ {\tilde{\varPi }_{SC} } \right]^{*}$ can be obtained as follows

Case 1 $$p \in \left( {c_{m} + c_{r} + \Delta_{1} ,2\left( {c_{m} + c_{r} } \right) - v - 2\Delta_{2} } \right]$$

23 $$E\left[ {\tilde{\varPi }_{SC} } \right]^{*} = \frac{1}{2}\int_{{{\kern 1pt} 0}}^{{{\kern 1pt} L\left( {q^{*} } \right)}} {\left( {\tilde{p}_{L}^{ - 1} \left( \alpha \right) - \tilde{v}_{R}^{ - 1} \left( \alpha \right)} \right)L^{ - 1} \left( \alpha \right)} \text{d} \alpha .$$

where $L\left( {q^{*} } \right)$ is given as in Eq. (19).

Case 2 $$p \in \left[ {2\left( {c_{m} + c_{r} } \right) - v - 2\Delta_{2} , + \infty } \right)$$

24 $$E\left[ {\tilde{\varPi }_{SC} } \right]^{*} = - \frac{1}{2}\int_{{{\kern 1pt} 0}}^{{{\kern 1pt} R\left( {q^{*} } \right)}} {\left( {\tilde{p}_{R}^{ - 1} \left( \alpha \right) - \tilde{v}_{L}^{ - 1} \left( \alpha \right)} \right)} R^{ - 1} \left( \alpha \right)\text{d} \alpha + E\left[ {\tilde{p}\tilde{D}} \right] - \frac{1}{2}\int_{0}^{1} {\left( {\tilde{v}_{L}^{ - 1} \left( \alpha \right)R^{ - 1} \left( \alpha \right) + \tilde{v}_{R}^{ - 1} \left( \alpha \right)L^{ - 1} \left( \alpha \right)} \right)} \text{d} \alpha .$$

where $R\left( {q^{*} } \right)$ is given as in Eq. (21).

In the next subsections, we develop two types of supply chain coordinating contracts, namely, the revenue sharing contract and the return contract that provide incentive mechanism to the retailer and the manufacturer.

### Revenue sharing contract

In the RS contract, the manufacturer shares a portion of revenue for retailer. Let $\varPhi$ denote the percentage revenue the retailer keeps, and then $1 - \varPhi$ denotes the portion the manufacturer shares, where $0 < \varPhi < 1$.

Thus, the fuzzy profit functions of the manufacturer and retailer are

25 $$\tilde{\varPi }_{M} = \left( {1 - \varPhi } \right)\left( {\tilde{p}\hbox{min} \left( {q,\tilde{D}} \right) + \tilde{v}\hbox{max} \left( {q - \tilde{D},0} \right)} \right) + wq - \tilde{c}_{s} q,$$

26 $$\tilde{\varPi }_{R} = \varPhi \left( {\tilde{p}\hbox{min} \left( {q,\tilde{D}} \right) + \tilde{v}\hbox{max} \left( {q - \tilde{D},0} \right)} \right) - wq - \tilde{c}_{r} q.$$

The retailer wants to get the optimal order quantity $q$ which maximizes his fuzzy expected profit $E\left[ {\tilde{\varPi }_{R} } \right]$. Thus, the retailer’s optimal objective function is

$$\text{Max}_{q} E\left[ {\tilde{\varPi }_{R} } \right] = E\left[ {\varPhi \left( {\tilde{p}\hbox{min} \left( {q,\tilde{D}} \right) + \tilde{v}\hbox{max} \left( {q - \tilde{D},0} \right)} \right) - wq - \tilde{c}_{r} q} \right]$$

27 $$\text{s} .t.\quad {\kern 1pt} d_{1} \le q \le d_{3} .$$

#### **Theorem 5**

*In the RS contract, the optimal wholesale price*$w^{*}$*is*

28 $$w^{*} = \varPhi \left( {c_{m} + c_{r} - 0.25\Delta_{31} + 0.25\Delta_{32} - 0.25\Delta_{41} + 0.25\Delta_{42} } \right) - \left( {c_{r} - 0.25\Delta_{41} + 0.25\Delta_{42} } \right).$$

#### *Proof*

Case 1 $$q \in \left[ {d_{1} ,d_{2} } \right]$$

Similar to the discussion in Theorem 1, the fuzzy expected profit $E\left[ {\tilde{\varPi }_{R} } \right]$ in this case is

29 $$E\left[ {\tilde{\varPi }_{R} } \right] = - \frac{\varPhi }{2}\int_{{{\kern 1pt} 0}}^{{{\kern 1pt} L\left( q \right)}} {\left( {\tilde{p}_{L}^{ - 1} \left( \alpha \right) - \tilde{v}_{R}^{ - 1} \left( \alpha \right)} \right)} \left( {q - L^{ - 1} \left( \alpha \right)} \right)\text{d} \alpha + \left( {\varPhi E\left[ {\tilde{p}} \right] - E\left[ {\tilde{c}_{r} } \right] - w} \right)q.$$

The first-order and second-order derivatives $E\left[ {\tilde{\varPi }_{R} } \right]$ about $q$ are

$$\begin{aligned}& \frac{{\text{d} E\left[ {\tilde{\varPi }_{R} } \right]}}{{\text{d} q}} = - \frac{\varPhi }{2}\int_{{{\kern 1pt} 0}}^{{{\kern 1pt} L\left( q \right)}} {\left( {\tilde{p}_{L}^{ - 1} \left( \alpha \right) - \tilde{v}_{R}^{ - 1} \left( \alpha \right)} \right)} \text{d} \alpha + \varPhi E\left[ {\tilde{p}} \right] - E\left[ {\tilde{c}_{m} } \right] - w, \hfill \\ &\frac{{\text{d}^{2} E\left[ {\tilde{\varPi }_{R} } \right]}}{{\text{d} q^{2} }} = - \frac{\varPhi }{2}\left( {\tilde{p}_{L}^{ - 1} \left( {L\left( q \right)} \right) - \tilde{v}_{R}^{ - 1} \left( {L\left( q \right)} \right)} \right)L^{{\prime }} \left( q \right). \hfill \\ \end{aligned}$$

Since $L\left( q \right)$ is an increasing function about $q$ with $L^{{\prime }} \left( q \right) > 0$, therefore $\frac{{\text{d}^{2} E\left[ {\tilde{\varPi }_{R} } \right]}}{{\text{d} q^{2} }} < 0$ and $E\left[ {\tilde{\varPi }_{R} } \right]$ is a concave function with respect to $q$ for $q \in \left[ {d_{1} ,d_{2} } \right].$

Hence, let $$\frac{{\text{d} E\left[ {\tilde{\varPi }_{R} } \right]}}{{\text{d} q}} = 0$$, we can derive the optimal order quantity $$q^{**}$$ in the RS contract as

$$- \frac{\varPhi }{2}\int_{{{\kern 1pt} 0}}^{{{\kern 1pt} L\left( {q^{**} } \right)}} {\left( {\tilde{p}_{L}^{ - 1} \left( \alpha \right) - \tilde{v}_{R}^{ - 1} \left( \alpha \right)} \right)} \text{d} \alpha + \varPhi E\left[ {\tilde{p}} \right] - E\left[ {\tilde{c}_{m} } \right] - w = 0.$$

That is

30 $$\frac{1}{2}\int_{{{\kern 1pt} 0}}^{{{\kern 1pt} L\left( {q^{**} } \right)}} {\left( {\tilde{p}_{L}^{ - 1} \left( \alpha \right) - \tilde{v}_{R}^{ - 1} \left( \alpha \right)} \right)\text{d} \alpha } = E\left[ {\tilde{p}} \right] - \frac{{w + E\left[ {\tilde{c}_{r} } \right]}}{\varPhi }.$$

For coordinating this supply chain, $$q^{**} = q^{*}$$ must hold. This means that the optimal order in the RS contract is the same as that in fuzzy centralized decision-making system.

Comparing Eq. (30) with Eq. (13), we have

$$\begin{aligned} w^{*} & = \varPhi \left( {E\left[ {\tilde{c}_{m} } \right] + E\left[ {\tilde{c}_{r} } \right]} \right) - E\left[ {\tilde{c}_{r} } \right] \\ & = \varPhi \left( {c_{m} + c_{r} - 0.25\Delta_{31} + 0.25\Delta_{32} - 0.25\Delta_{41} + 0.25\Delta_{42} } \right) - \left( {c_{r} - 0.25\Delta_{41} + 0.25\Delta_{42} } \right). \\ \end{aligned}$$

Case 2 $$q \in \left( {d_{2} ,d_{3} } \right]$$

Similar to the discussion in Theorem 2, the fuzzy expected profit $E\left[ {\tilde{\varPi }_{R} } \right]$ in this case is

31 $$\begin{aligned}E\left[ {\tilde{\varPi }_{R} } \right] &= \frac{\varPhi }{2}\int_{{{\kern 1pt} 0}}^{{{\kern 1pt} R\left( q \right)}} {\left( {\tilde{p}_{R}^{ - 1} \left( \alpha \right) - \tilde{v}_{l}^{ - 1} \left( \alpha \right)} \right)\left( {q - R^{ - 1} \left( \alpha \right)} \right)} \text{d} \alpha + \left( {\varPhi E\left[ {\tilde{v}} \right] - E\left[ {\tilde{c}_{r} } \right] - w} \right)q \\&\quad+\, \varPhi E\left[ {\tilde{p}\tilde{D}} \right] - \frac{\varPhi }{2}\int_{{{\kern 1pt} 0}}^{{{\kern 1pt} 1}} {\left( {\tilde{v}_{L}^{ - 1} \left( \alpha \right)R^{ - 1} \left( \alpha \right) + \tilde{v}_{R}^{ - 1} \left( \alpha \right)L^{ - 1} \left( \alpha \right)} \right)} \text{d} \alpha . \end{aligned}$$

The first-order and second-order derivatives $E\left[ {\tilde{\varPi }_{R} } \right]$ about $q$ are

$$\begin{aligned} & \frac{{\text{d} E\left[ {\tilde{\varPi }_{R} } \right]}}{{\text{d} q}} = \frac{\varPhi }{2}\int_{{{\kern 1pt} 0}}^{{{\kern 1pt} R\left( q \right)}} {\left( {\tilde{p}_{R}^{ - 1} \left( \alpha \right) - \tilde{v}_{L}^{ - 1} \left( \alpha \right)} \right)} \text{d} \alpha + \varPhi E\left[ {\tilde{v}} \right] - E\left[ {\tilde{c}_{r} } \right] - w, \\ & \frac{{\text{d}^{2} E\left[ {\tilde{\varPi }_{R} } \right]}}{{\text{d} q^{2} }} = \frac{\varPhi }{2}\left( {\tilde{p}_{R}^{ - 1} \left( {R\left( q \right)} \right) - \tilde{v}_{L}^{ - 1} \left( {R\left( q \right)} \right)} \right)R^{{\prime }} \left( q \right). \\ \end{aligned}$$

Since $R\left( q \right)$ is a decreasing function about $q$ with $R^{\;'} \left( q \right) < 0$, therefore $\frac{{\text{d}^{2} E\left[ {\tilde{\varPi }_{R} } \right]}}{{\text{d} q^{2} }} < 0$ and $E\left[ {\tilde{\varPi }_{R} } \right]$ is a concave function with respect to $q$ for $q \in \left( {d_{2} ,d_{3} } \right].$

Hence, let $$\frac{{\text{d} E\left[ {\tilde{\varPi }_{R} } \right]}}{{\text{d} q}} = 0$$, we can derive the optimal order quantity $$q^{**}$$ as

$$\frac{\varPhi }{2}\int_{{{\kern 1pt} 0}}^{{{\kern 1pt} R\left( {q^{**} } \right)}} {\left( {\tilde{p}_{R}^{ - 1} \left( \alpha \right) - \tilde{v}_{L}^{ - 1} \left( \alpha \right)} \right)} \text{d} \alpha + \varPhi E\left[ {\tilde{v}} \right] - E\left[ {\tilde{c}_{r} } \right] - w = 0.$$

That is

32 $$\frac{1}{2}\int_{0}^{{R\left( {q^{**} } \right)}} {\left( {\tilde{p}_{R}^{ - 1} \left( \alpha \right) - \tilde{v}_{L}^{ - 1} \left( \alpha \right)} \right)} \text{d} \alpha = \frac{{w + E\left[ {\tilde{c}_{r} } \right]}}{\varPhi } - E\left[ {\tilde{v}} \right].$$

For coordinating this supply chain, $$q^{**} = q^{*}$$ must hold.

Comparing Eq. (32) with Eq. (16), we have

$$\begin{aligned} w^{*} & = \varPhi \left( {E\left[ {\tilde{c}_{m} } \right] + E\left[ {\tilde{c}_{r} } \right]} \right) - E\left[ {\tilde{c}_{r} } \right] \\ & = \varPhi \left( {c_{m} + c_{r} - 0.25\Delta_{31} + 0.25\Delta_{32} - 0.25\Delta_{41} + 0.25\Delta_{42} } \right) - \left( {c_{r} - 0.25\Delta_{41} + 0.25\Delta_{42} } \right). \\ \end{aligned}$$

Theorem 5 is proved.

#### **Proposition 7**

*If*$\Delta_{31} = \Delta_{32} = \Delta_{41} = \Delta_{42} = 0$*, then the fuzzy parameters*$\tilde{c}_{m}$*and*$\tilde{c}_{r}$*degenerate to the crisp values, and the solution in Theorem 5 degenerates to*

33 $$w^{*} = \varPhi \left( {c_{m} + c_{r} } \right) - c_{r} .$$

The solution in Proposition 7 is the same as the result in Cachon and Lariviere (2005), they studied the RS contract in a random demand environment. It shows that the optimal wholesale price is not affected with the change of the fuzziness of demand

#### **Theorem 6**

*In the RS contract,the optimal fuzzy expected profit functions of the members are*

34 $$E\left[ {\tilde{\varPi }_{R} } \right]^{*} = \varPhi E\left[ {\tilde{\varPi }_{SC} } \right]^{*} ,\quad {\text{and}}\quad E\left[ {\tilde{\varPi }_{M} } \right]^{*} = \left( {1 - \varPhi } \right)E\left[ {\tilde{\varPi }_{SC} } \right]^{*} .$$

#### *Proof*

Case 1 $$q \in \left[ {d_{1} ,d_{2} } \right]$$

Substituting $$w^{*}$$ in Eq. (28) and $$q^{**} = q^{*}$$ into Eq. (29), the optimal fuzzy profit $$E\left[ {\tilde{\varPi }_{R} } \right]^{*}$$ in the RS contract is

$$\begin{aligned} E\left[ {\tilde{\varPi }_{R} } \right]^{*} & = - \frac{\varPhi }{2}\int_{{{\kern 1pt} 0}}^{{{\kern 1pt} L\left( {q^{*} } \right)}} {\left( {\tilde{p}_{L}^{ - 1} \left( \alpha \right) - \tilde{v}_{R}^{ - 1} \left( \alpha \right)} \right)L^{ - 1} \left( \alpha \right)} \text{d} \alpha \\ & = \varPhi E\left[ {\tilde{\varPi }_{SC} } \right]^{*} . \\ \end{aligned}$$

Then, the fuzzy expected profit $E\left[ {\tilde{\varPi }_{M} } \right]^{*}$ in this case is

$$\begin{aligned} E\left[ {\tilde{\varPi }_{M} } \right]^{*} & = E\left[ {\tilde{\varPi }_{SC} } \right]^{*} - E\left[ {\tilde{\varPi }_{R} } \right]^{*} \\ & = \left( {1 - \varPhi } \right)E\left[ {\tilde{\varPi }_{SC} } \right]^{*} . \\ \end{aligned}$$

Case 2 $$q \in \left( {d_{2} ,d_{3} } \right]$$

Substituting $$w^{*}$$ in Eq. (30) and $$q^{**} = q^{*}$$ into Eq. (31), the optimal fuzzy profit $$E\left[ {\tilde{\varPi }_{R} } \right]^{*}$$ in the RS contract is

$$\begin{aligned} E\left[ {\tilde{\varPi }_{R} } \right]^{*} & = - \frac{\varPhi }{2}\int_{{{\kern 1pt} 0}}^{{{\kern 1pt} R\left( {q^{*} } \right)}} {\left( {\tilde{p}_{R}^{ - 1} \left( \alpha \right) - \tilde{v}_{L}^{ - 1} \left( \alpha \right)} \right)} R^{ - 1} \left( \alpha \right)\text{d} \alpha + \varPhi E\left[ {\tilde{p}\tilde{D}} \right] - \frac{\varPhi }{2}\int_{{{\kern 1pt} 0}}^{{{\kern 1pt} 1}} {\left( {\tilde{v}_{L}^{ - 1} \left( \alpha \right)R^{ - 1} \left( \alpha \right) + \tilde{v}_{R}^{ - 1} \left( \alpha \right)L^{ - 1} \left( \alpha \right)} \right)} \text{d} \alpha \\ & = \varPhi E\left[ {\tilde{\varPi }_{SC} } \right]^{*} . \\ \end{aligned}$$

Then, the fuzzy expected profit $E\left[ {\tilde{\varPi }_{M} } \right]^{*}$ in this case is

$$\begin{aligned} E\left[ {\tilde{\varPi }_{M} } \right]^{*} & = E\left[ {\tilde{\varPi }_{SC} } \right]^{*} - E\left[ {\tilde{\varPi }_{R} } \right]^{*} \\ & = \left( {1 - \varPhi } \right)E\left[ {\tilde{\varPi }_{SC} } \right]^{*} . \\ \end{aligned}$$

Theorem 6 is proved.

In Theorem 6, the parameter $\varPhi$ depends on the negotiating ability for the retailer and manufacturer. It shows that, the more powerful bargaining the retailer has, the more fuzzy expected profit he will derive.

### Return contract

In the return contract, if the retailer has the unsold products, then the manufacturer should pay unit return price *b* of these products for the retailer. Thus, the fuzzy profit of the manufacturer and retailer can be expressed as follows respectively

35 $$\tilde{\varPi }_{s} = wq - \tilde{c}_{s} q - b\hbox{max} \left( {q - \tilde{D},0} \right) + \tilde{v}\hbox{max} \left( {q - \tilde{D},0} \right),$$

36 $$\tilde{\varPi }_{R} = \tilde{p}\hbox{min} \left( {q,\tilde{D}} \right) + b\hbox{max} \left( {q - \tilde{D},0} \right) - \tilde{c}_{r} q - wq.$$

In the return contract, the retailer wants to get the optimal order quantity $q$ which maximizes his fuzzy expected profit $E\left[ {\tilde{\varPi }_{R} } \right]$, which can be denoted as the following model

$$\text{Max}_{q} E\left[ {\tilde{\varPi }_{R} } \right] = E\left[ {\tilde{p}\hbox{min} \left( {q,\tilde{D}} \right) + b\hbox{max} \left( {q - \tilde{D},0} \right) - \tilde{c}_{r} q - wq} \right]$$

37 $$\text{s} .t.\quad {\kern 1pt} d_{1} \le q \le d_{3} .$$

#### **Theorem 7**

*When*$c_{m} + c_{r} + \Delta_{1} < p \le 2\left( {c_{m} + c_{r} } \right) - v - 2\Delta_{2}$*, the optimal return price*$b^{*}$*in return contract is*

38 $$\begin{aligned} b^{*} & = \frac{{w - c_{m} + 0.25\Delta_{31} - 0.25\Delta_{32} }}{{p - c_{m} - c_{r} - \Delta_{1} }}\left( {\sqrt {0.25\left( {p - v - \Delta_{11} - \Delta_{22} } \right)^{2} + \left( {\Delta_{11} + \Delta_{22} } \right)\left( {p - c_{m} - c_{r} - \Delta_{1} } \right)} + 0.5\left( {p - v - \Delta_{11} - \Delta_{22} } \right)} \right) \\ & \quad - \frac{{\Delta_{22} }}{{\Delta_{11} + \Delta_{22} }}\left( {\sqrt {0.25\left( {p - v - \Delta_{11} - \Delta_{22} } \right)^{2} + \left( {\Delta_{11} + \Delta_{22} } \right)\left( {p - c_{m} - c_{r} - \Delta_{1} } \right)} - 0.5\left( {p - v - \Delta_{11} - \Delta_{22} } \right)} \right) + v + \Delta_{22} . \\ \end{aligned}$$

#### *Proof*

If $q \in \left[ {d_{1} ,d_{2} } \right]$, similar to the discussion in Theorem 1, the fuzzy expected profit $E\left[ {\tilde{\varPi }_{R} } \right]$ in this case is

39 $$E\left[ {\tilde{\varPi }_{R} } \right] = \frac{1}{2}\int_{{{\kern 1pt} 0}}^{{{\kern 1pt} L\left( q \right)}} {\left( {\tilde{p}_{L}^{ - 1} \left( \alpha \right)L^{ - 1} \left( \alpha \right) - \tilde{p}_{R}^{ - 1} \left( \alpha \right)q - bL^{ - 1} \left( \alpha \right)} \right)} \text{d} \alpha + \frac{1}{2}bL\left( q \right)q + E\left[ {\tilde{p}} \right]q - E\left[ {\tilde{c}_{r} } \right]q - wq.$$

The first-order and second-order derivatives $E\left[ {\tilde{\varPi }_{R} } \right]$ about $q$ are

$$\begin{aligned} & \frac{{\text{d} E\left[ {\tilde{\varPi }_{R} } \right]}}{{\text{d} q}} = - \frac{1}{2}\int_{{{\kern 1pt} 0}}^{{{\kern 1pt} L\left( q \right)}} {\tilde{p}_{L}^{ - 1} } \left( \alpha \right)\text{d} \alpha + \frac{1}{2}bL\left( q \right) + E\left[ {\tilde{p}} \right] - E\left[ {\tilde{c}_{r} } \right] - w, \\ & \frac{{\text{d}^{2} E\left[ {\tilde{\varPi }_{R} } \right]}}{{\text{d} q^{2} }} = - \frac{1}{2}\left( {\tilde{p}_{L}^{ - 1} \left( {L\left( q \right)} \right) - b} \right)L^{{\prime }} \left( q \right). \\ \end{aligned}$$

Since $L\left( q \right)$ is an increasing function about $q$ with $L^{{\prime }} \left( q \right) > 0$, and for any given $L\left( q \right) \in \left[ {0,1} \right]$, $\tilde{p}_{L}^{ - 1} \left( {L\left( q \right)} \right) > b$, therefore $\frac{{\text{d}^{2} E\left[ {\tilde{\varPi }_{R} } \right]}}{{\text{d} q^{2} }} < 0$ and $E\left[ {\tilde{\varPi }_{R} } \right]$ is a concave function with respect to $q$ for $q \in \left[ {d_{1} ,d_{2} } \right].$

Hence, let $$\frac{{\text{d} E\left[ {\tilde{\varPi }_{R} } \right]}}{{\text{d} q}} = 0$$, we can derive the optimal order quantity $$q^{***}$$ in the return contract as

$$- \frac{1}{2}\int_{{{\kern 1pt} 0}}^{{{\kern 1pt} L\left( {q^{***} } \right)}} {\tilde{p}_{L}^{ - 1} } \left( \alpha \right)\text{d} \alpha + \frac{1}{2}bL\left( {q^{***} } \right) + E\left[ {\tilde{p}} \right] - E\left[ {\tilde{c}_{r} } \right] - w = 0.$$

That is

40 $$\frac{1}{2}\int_{{{\kern 1pt} 0}}^{{{\kern 1pt} L\left( {q^{***} } \right)}} {\tilde{p}_{L}^{ - 1} } \left( \alpha \right)\text{d} \alpha = \frac{1}{2}bL\left( {q^{***} } \right) + E\left[ {\tilde{p}} \right] - E\left[ {\tilde{c}_{r} } \right] - w.$$

For coordinating this supply chain, $$q^{***} = q^{*}$$ must hold. That is to say, the optimal order quantity $$q^{***}$$ chosen by the retailer in return contract is equal to that in centralized decision-making system.

Comparing Eq. (40) with Eq. (13), we have

41 $$\begin{aligned} b^{*} & = \frac{{2\left( {w - E\left[ {\tilde{c}_{m} } \right]} \right) + \int_{{{\kern 1pt} 0}}^{{{\kern 1pt} L\left( {q^{*} } \right)}} {\tilde{v}_{R}^{ - 1} \left( \alpha \right)\text{d} \alpha } }}{{L\left( {q^{*} } \right)}} \\ & = \frac{{2\left( {w - c_{m} + 0.25\Delta_{31} - 0.25\Delta_{32} } \right)}}{{L\left( {q^{*} } \right)}} - 0.5\Delta_{22} L\left( {q^{*} } \right) + v + \Delta_{22} . \\ \end{aligned}$$

Using Eq. (20), we can get the optimal return price $b^{*}$ as shown in Eq. (38).

Theorem 7 is proved.

#### **Theorem 8**

*When*$p \ge 2\left( {c_{m} + c_{r} } \right) - v - 2\Delta_{2}$*, the optimal return price*$b^{*}$*in return contract is*

42 $$\begin{aligned} b^{*} & = \frac{{w - c_{m} - 0.25\Delta_{21} + 0.25\Delta_{22} + 0.25\Delta_{31} - 0.25\Delta_{32} }}{{p - c_{m} - c_{r} - \Delta_{3} }}\left( {0.5\left( {p - v - \Delta_{12} - \Delta_{21} } \right) + \sqrt {0.25\left( {p - v + \Delta_{12} + \Delta_{21} } \right)^{2} - \left( {\Delta_{12} + \Delta_{21} } \right)\left( {c_{m} + c_{r} - v + \Delta_{3} } \right)} } \right) \\ & \quad + \frac{{\Delta_{21} }}{{\Delta_{12} + \Delta_{21} }}\left( {0.5\left( {p - v + \Delta_{12} + \Delta_{21} } \right) - \sqrt {0.25\left( {p - v + \Delta_{12} + \Delta_{21} } \right)^{2} - \left( {\Delta_{12} + \Delta_{21} } \right)\left( {c_{m} + c_{r} - v + \Delta_{3} } \right)} } \right) + v. \\ \end{aligned}$$

#### *Proof*

If $q \in \left( {d_{2} ,d_{3} } \right]$, similar to the discussion in Theorem 2, the fuzzy expected profit $E\left[ {\tilde{\varPi }_{R} } \right]$ in this case is

43 $$E\left[ {\tilde{\varPi }_{R} } \right] = \frac{1}{2}\int_{{{\kern 1pt} 0}}^{{{\kern 1pt} R\left( q \right)}} {\left( {\tilde{p}_{R}^{ - 1} \left( \alpha \right)q + bR^{ - 1} \left( \alpha \right) - \tilde{p}_{R}^{ - 1} \left( \alpha \right)R^{ - 1} \left( \alpha \right)} \right)} \text{d} \alpha + bq - \frac{1}{2}bR\left( q \right)q - wq - E\left[ {\tilde{c}_{r} } \right]q + E\left[ {\tilde{p}\tilde{D}} \right] - bE\left[ {\tilde{D}} \right].$$

The first-order and second-order derivatives $E\left[ {\tilde{\varPi }_{R} } \right]$ about $q$ are

$$\begin{aligned} & \frac{{\text{d} E\left[ {\tilde{\varPi }_{R} } \right]}}{{\text{d} q}} = \frac{1}{2}\int_{{{\kern 1pt} 0}}^{{{\kern 1pt} R\left( q \right)}} {\tilde{p}_{R}^{ - 1} } \left( \alpha \right)\text{d} \alpha - \frac{1}{2}bR\left( q \right) + b - w - E\left[ {\tilde{c}_{r} } \right], \\ & \frac{{\text{d}^{2} E\left[ {\tilde{\varPi }_{R} } \right]}}{{\text{d} q^{2} }} = \frac{1}{2}\left( {\tilde{p}_{R}^{ - 1} \left( {R\left( q \right)} \right) - b} \right)R^{{\prime }} \left( q \right). \\ \end{aligned}$$

Since $R\left( q \right)$ is a decreasing function about $q$ with $R^{{\prime }} \left( q \right) < 0$ and for any given $R\left( q \right) \in \left[ {0,1} \right]$, $\tilde{p}_{R}^{ - 1} \left( {R\left( q \right)} \right) > b$, therefore $\frac{{\text{d}^{2} E\left[ {\tilde{\varPi }_{R} } \right]}}{{\text{d} q^{2} }} < 0$ and $E\left[ {\tilde{\varPi }_{R} } \right]$ is a concave function with respect to $q$ for $q \in \left( {d_{2} ,d_{3} } \right].$

Hence, let $$\frac{{\text{d} E\left[ {\tilde{\varPi }_{R} } \right]}}{{\text{d} q}} = 0$$, we can derive the optimal order quantity $$q^{***}$$ in the return contract as

$$\frac{1}{2}\int_{{{\kern 1pt} 0}}^{{{\kern 1pt} R\left( {q^{***} } \right)}} {\tilde{p}_{R}^{ - 1} } \left( \alpha \right)\text{d} \alpha - \frac{1}{2}bR\left( {q^{***} } \right) + b - w - E\left[ {\tilde{c}_{r} } \right] = 0.$$

That is

44 $$\frac{1}{2}\int_{{{\kern 1pt} 0}}^{{{\kern 1pt} R\left( {q^{***} } \right)}} {\tilde{p}_{R}^{ - 1} } \left( \alpha \right)\text{d} \alpha = \frac{1}{2}bR\left( {q^{***} } \right) - b + w + E\left[ {\tilde{c}_{r} } \right].$$

For coordinating this supply chain, $$q^{***} = q^{*}$$ must hold.

Comparing Eq. (44) with Eq. (16), we have

$$\begin{aligned} b^{*} & = \frac{{w - E\left[ {\tilde{c}_{m} } \right] + E\left[ {\tilde{v}} \right] - \tfrac{1}{2}\int_{{{\kern 1pt} 0}}^{{{\kern 1pt} R\left( {q^{*} } \right)}} {\tilde{v}_{L}^{ - 1} \left( \alpha \right)\text{d} \alpha } }}{{1 - 0.5R\left( {q^{*} } \right)}} \\ & = \frac{{w - c_{m} - 0.25\Delta_{21} + 0.25\Delta_{22} + 0.25\Delta_{31} - 0.25\Delta_{32} }}{{1 - 0.5R\left( {q^{*} } \right)}} + 0.5\Delta_{21} R\left( {q^{*} } \right) + v. \\ \end{aligned}$$

Using Eq. (21), we can get the optimal return price $b^{*}$ as shown in Eq. (42).

Theorem 8 is proved.

#### **Proposition 8**

*If*$\Delta_{ij} \to 0$, $i = 1,2,3,4$, $j = 1,2$, *then the fuzzy parameters*$\tilde{p}$, $\tilde{v}$, $\tilde{c}_{m}$, $\tilde{c}_{r}$*degenerate to the crisp values, and the results in Theorems 7 and 8 degenerate to*

45 $$b^{*} = \frac{{\left( {p - v} \right)\left( {w - c_{m} } \right)}}{{p - c_{m} - c_{r} }} + v.$$

*There are just the results in a fuzzy demand environment.*

#### *Proof*

Similar to the proof of Proposition 6.

#### **Proposition 9**

*If we do not take the fuzzy salvage value of product*$\tilde{v}$*and the retailer’s fuzzy operational cost of product*$\tilde{c}_{r}$*into consideration, and let*$\Delta_{31} = \Delta_{32} = 0$*, then the results of Theorems 7 and 8 reduce to*

46 $$b^{*} = \left\{ {\begin{array}{*{20}l} {\frac{{\left( {w - c_{m} } \right)\left( {\sqrt {0.25p^{2} + \Delta_{11} \left( {0.5p - c_{m} + 0.25\Delta_{12} } \right)} + 0.5\left( {p - \Delta_{11} } \right)} \right)}}{{p - c_{m} - 0.25\Delta_{11} + 0.25\Delta_{12} }},} \hfill &\quad {p \le 2c_{m} - 0.5\Delta_{12} ,} \hfill \\ {\frac{{\left( {w - c_{m} } \right)\left( {\sqrt {0.25\left( {p + \Delta_{12} } \right)^{2} - \Delta_{12} c_{m} } + 0.5\left( {p - \Delta_{12} } \right)} \right)}}{{p - c_{m} }},} \hfill & \quad{p > 2c_{m} - 0.5\Delta_{12} .} \hfill \\ \end{array} } \right.$$

*There are just the solutions studied in* Yu and Jin (2011).

From Eqs. (19), (21), (39), (40), (43) and (44), the optimal fuzzy expected profits $$E\left[ {\tilde{\varPi }_{R} } \right]^{**}$$ and $$E\left[ {\tilde{\varPi }_{M} } \right]^{**}$$ can be derived as

Case 1 $$c_{m} + c_{r} + \Delta_{1} < p \le 2\left( {c_{m} + c_{r} } \right) - v - 2\Delta_{2}$$

47 $$E\left[ {\tilde{\varPi }_{R} } \right]^{**} = \frac{1}{2}\int_{{{\kern 1pt} 0}}^{{{\kern 1pt} L\left( {q^{*} } \right)}} {\left( {\tilde{p}_{L}^{ - 1} \left( \alpha \right) - b^{*} } \right)L^{ - 1} \left( \alpha \right)} \text{d} \alpha ,$$

48 $$E\left[ {\tilde{\varPi }_{M} } \right]^{**} = \frac{1}{2}\int_{{{\kern 1pt} 0}}^{{{\kern 1pt} L\left( {q^{*} } \right)}} {\left( {b^{*} - \tilde{v}_{R}^{ - 1} \left( \alpha \right)} \right)L^{ - 1} \left( \alpha \right)} \text{d} \alpha .$$

where $L\left( {q^{*} } \right)$ and $b^{*}$ are given as in Eqs. (19) and (38) respectively.

Case 2 $$p \ge 2\left( {c_{m} + c_{r} } \right) - v - 2\Delta_{2}$$

49 $$E\left[ {\tilde{\varPi }_{R} } \right]^{**} = - \frac{1}{2}\int_{{{\kern 1pt} 0}}^{{{\kern 1pt} R\left( {q^{*} } \right)}} {\left( {\tilde{p}_{R}^{ - 1} \left( \alpha \right) - b^{*} } \right)} R^{ - 1} \left( \alpha \right)\text{d} \alpha - b^{*} E\left[ {\tilde{D}} \right] + E\left[ {\tilde{p}\tilde{D}} \right],$$

50 $$E\left[ {\tilde{\varPi }_{M} } \right]^{**} = - \frac{1}{2}\int_{{{\kern 1pt} 0}}^{{{\kern 1pt} R\left( {q^{*} } \right)}} {\left( {b^{*} - \tilde{v}_{L}^{ - 1} \left( \alpha \right)} \right)R^{ - 1} \left( \alpha \right)} \text{d} \alpha + b^{*} E\left[ {\tilde{D}} \right] - \frac{1}{2}\int_{{{\kern 1pt} 0}}^{{{\kern 1pt} 1}} {\left( {\tilde{v}_{L}^{ - 1} \left( \alpha \right)R^{ - 1} \left( \alpha \right) + \tilde{v}_{R}^{ - 1} \left( \alpha \right)L^{ - 1} \left( \alpha \right)} \right)} \text{d} \alpha .$$

where $R\left( {q^{*} } \right)$ and $b^{*}$ are given as in Eqs. (21) and (42) respectively.

## Numerical examples

Because the optimal solutions obtained above are in complicated forms, we have to provide some numerical examples to further illustrate the effectives of the proposed models.

### Discussion 1

In this subsection, we discuss the impact of the fuzziness of parameter $$\tilde{D}$$ on the optimal solutions in both contracts. The fuzzy salvage value of product $$\tilde{v}$$, the fuzzy production cost of product $$\tilde{c}_{m}$$ and the fuzzy operational cost of product $$\tilde{c}_{r}$$ estimated by the experience of the decision maker are assumed to be the triangular fuzzy numbers. The salvage value of item is nearly $ 3, but not greater than $ 4 and not less than 2, that is $$\tilde{v} = \left( {2,3,4} \right)$$. Similarly, the production cost of item by the manufacturer is nearly $ 15, but not greater than $ 16 and not less than 14, that is $$\tilde{c}_{m} = \left( {14,15,16} \right)$$. The operational cost of item by the retailer is nearly $ 2, but not greater than $ 3 and not less than 1, that is $$\tilde{c}_{r} = \left( {1,2,3} \right)$$.

Since the optimal order quantity $q^{*}$ has two cases, the optimal policies in two contracts can be listed in Tables 1 and 2.

**Table 1:** The RS contract policies with $\varPhi = 0.6$

$\tilde{p}$	$\tilde{D}$	$q^{*}$	$w^{*}$	$E\left[ {\tilde{\varPi }_{R} } \right]^{*}$	$E\left[ {\tilde{\varPi }_{M} } \right]^{*}$	$E\left[ {\tilde{\varPi }_{SC} } \right]^{*}$
(22, 25, 28)	(100, 200, 300)	181.51	8.20	681.03	454.02	1135.05
	(110, 200, 290)	183.36	8.20	708.93	472.62	1181.55
	(120, 200, 280)	185.21	8.20	736.82	491.22	1228.04
	(130, 200, 270)	187.06	8.20	764.72	509.82	1274.54
	(140, 200, 260)	188.90	8.20	792.62	528.41	1321.03
	(150, 200, 250)	190.75	8.20	820.52	547.01	1367.53
(32, 35, 38)	(100, 200, 300)	218.54	8.20	1736.75	1157.83	2894.58
	(110, 200, 290)	216.68	8.20	1779.07	1186.05	2965.12
	(120, 200, 280)	214.83	8.20	1821.40	1214.26	3035.66
	(130, 200, 270)	212.97	8.20	1863.72	1242.48	3106.20
	(140, 200, 260)	211.12	8.20	1906.05	1270.70	3176.75
	(150, 200, 250)	209.27	8.20	1948.37	1298.92	3247.29

**Table 2:** The return contract policies with $w = 20$

$$\tilde{p}$$	$$\tilde{D}$$	$$q^{*}$$	$$b^{*}$$	$$E\left[ {\tilde{\varPi }_{R} } \right]^{**}$$	$$E\left[ {\tilde{\varPi }_{M} } \right]^{**}$$	$$E\left[ {\tilde{\varPi }_{SC} } \right]^{*}$$
(22, 25, 28)	(100, 200, 300)	181.51	15.86	429.03	706.02	1135.05
	(110, 200, 290)	183.36	15.86	446.13	735.42	1181.55
	(120, 200, 280)	185.21	15.86	463.22	764.82	1228.04
	(130, 200, 270)	187.06	15.86	480.32	794.22	1274.54
	(140, 200, 260)	188.90	15.86	497.42	823.61	1321.03
	(150, 200, 250)	190.75	15.86	514.51	853.01	1367.53
(32, 35, 38)	(100, 200, 300)	218.54	11.84	2091.30	803.28	2894.58
	(110, 200, 290)	216.68	11.84	2142.17	822.95	2965.12
	(120, 200, 280)	214.83	11.84	2193.04	842.62	3035.66
	(130, 200, 270)	212.97	11.84	2243.91	862.29	3106.20
	(140, 200, 260)	211.12	11.84	2294.78	881.97	3176.75
	(150, 200, 250)	209.27	11.84	2345.65	901.64	3247.29

From Tables 1 and 2, we can get the results as follows

1. When $\tilde{p} = \left( {22,25,28} \right)$, that satisfies $p \le 2\left( {c_{m} + c_{r} } \right) - v - 2\Delta_{2}$, $q^{*}$ is smaller than the most possible value of the demand. When $\tilde{p} = \left( {32,35,38} \right)$, that satisfies $p > 2\left( {c_{m} + c_{r} } \right) - v - 2\Delta_{2}$, $q^{*}$ is larger than the most possible value of the demand in both contracts.
2. The fuzziness the parameter $\tilde{D}$ decreases as $d_{2}$ increases or $d_{3}$ decreases. No matter in what kind of supply chain contract, when $p \le 2\left( {c_{m} + c_{r} } \right) - v - 2\Delta_{2}$, the optimal order quantity $q^{*}$ increases as the fuzziness of parameter $\tilde{D}$ decreases. However, when $p > 2\left( {c_{m} + c_{r} } \right) - v - 2\Delta_{2}$, decreasing the fuzziness of parameter $\tilde{D}$ will decrease the order quantity $q^{*}$.
3. The change of retail price $\tilde{p}$ and fuzziness of parameter $\tilde{D}$ will not impact on the wholesale price $w^{*}$ in the RS contract. This is because the wholesale price $w^{*}$ is impacted only by the parameters $\tilde{c}_{s}$ and $\tilde{c}_{r}$. In addition, the change of the fuzziness of demand $\tilde{D}$ has no impact on the return price $b$ in the return contract.
4. When the fuzziness of market demand decreases, the expected profits for all members increase. In other words, the retailer, the manufacturer and the supply chain system all gain more expected profit when the fuzziness of demand is lower. This is intuitive because the lower the fuzziness of demand, the more efficient of the supply chain system. Therefore, for the manufacturer and retailer, they should seek as low fuzziness of market demand as possible in both contracts.

### Discussion 2

In this subsection, we discuss the impact of retail price’s fuzziness on optimal policies in two contracts. The demand of the market is nearly 200, but not greater than 300 and not less than 100, that is $\tilde{D} = \left( {100,200,300} \right)$. The other fuzzy parameters are the same as above discussion. The optimal solutions obtained are shown in Tables 3 and 4.

**Table 3:** The RS contract policies with $\varPhi = 0.6$

$\tilde{p}$	$q^{*}$	$w^{*}$	$E\left[ {\tilde{\varPi }_{R} } \right]^{*}$	$E\left[ {\tilde{\varPi }_{M} } \right]^{*}$	$E\left[ {\tilde{\varPi }_{SC} } \right]^{*}$
(20, 25, 30)	186.10	8.20	696.22	464.14	1160.36
(21, 25, 29)	183.79	8.20	688.46	458.97	1147.43
(22, 25, 28)	181.51	8.20	681.03	454.02	1135.05
(23, 25, 27)	179.25	8.20	673.94	449.29	1123.23
(24, 25, 26)	177.03	8.20	667.16	444.78	1111.94
(25, 25, 25)	174.86	8.20	660.70	440.47	1101.17
(30, 35, 40)	221.44	8.20	1762.66	1175.11	2937.77
(31, 35, 39)	220.00	8.20	1749.60	1166.40	2916.00
(32, 35, 38)	218.54	8.20	1736.75	1157.83	2894.58
(33, 35, 37)	217.05	8.20	1724.10	1149.40	2873.51
(34, 35, 36)	215.55	8.20	1711.68	1141.12	2852.80
(35, 35, 35)	214.03	8.20	1699.48	1132.99	2832.46

**Table 4:** The return contract policies with $w = 20$

$$\tilde{p}$$	$$q^{*}$$	$$b^{*}$$	$$E\left[ {\tilde{\varPi }_{R} } \right]^{**}$$	$$E\left[ {\tilde{\varPi }_{M} } \right]^{**}$$	$$E\left[ {\tilde{\varPi }_{SC} } \right]^{*}$$
(20, 25, 30)	186.10	15.18	442.45	717.91	1160.36
(21, 25, 29)	183.79	15.52	435.49	711.93	1147.43
(22, 25, 28)	181.51	15.86	429.03	706.02	1135.05
(23, 25, 27)	179.25	16.22	423.03	700.20	1123.23
(24, 25, 26)	177.03	16.60	417.45	694.49	1111.94
(25, 25, 25)	174.86	16.98	412.28	688.89	1101.17
(30, 35, 40)	221.44	11.63	2126.70	811.07	2937.77
(31, 35, 39)	220.00	11.73	2108.80	807.20	2916.00
(32, 35, 38)	218.54	11.84	2091.30	803.28	2894.58
(33, 35, 37)	217.05	11.96	2074.20	799.31	2873.51
(34, 35, 36)	215.55	12.08	2057.50	795.30	2852.80
(35, 35, 35)	214.03	12.20	2041.20	791.26	2832.46

5. The optimal order quantity $q^{*}$ drops as the fuzziness of retail price $\tilde{p}$ falls in both contracts. It indicates that increasing the fuzziness of retail price $\tilde{p}$ can simulate the order quantity.
6. The change of the fuzziness of retail price $\tilde{p}$ has no impact on $w^{*}$ in the RS contract. Conversely, the return price $b^{*}$ increases as the fuzziness of retail price $\tilde{p}$ decreases in the return contract.
7. When the fuzziness of retail price decreases, the expected profits for all members decrease. This is because a decrease in fuzziness of retail price results in a decrease in order quantity. This results in the decrease of the fuzzy expected profit of the retailer, the manufacturer and the whole supply chain. Therefore, for the manufacturer and the retailer, they should seek as high fuzziness of the retail price as possible in both contracts.

### Discussion 3

In this subsection, we discuss the impact of salvage value’s fuzziness on optimal policies in two contracts. The fuzzy demand is assumed to be $\tilde{D} = \left( {100,200,300} \right)$. The other fuzzy parameters are the same as above discussion. The optimal solutions obtained are shown in Tables 5 and 6.

**Table 5:** The RS contract policies with $\varPhi = 0.6$

$\tilde{p}$	$\tilde{v}$	$q^{*}$	$w^{*}$	$E\left[ {\tilde{\varPi }_{R} } \right]^{*}$	$E\left[ {\tilde{\varPi }_{M} } \right]^{*}$	$E\left[ {\tilde{\varPi }_{SC} } \right]^{*}$
(22, 25, 28)	(2.0, 3.0, 4.0)	181.51	8.20	681.03	454.02	1135.05
	(2.2, 3.0, 3.8)	181.05	8.20	679.59	453.06	1132.65
	(2.4, 3.0, 3.6)	180.60	8.20	678.16	452.10	1130.26
	(2.6, 3.0, 3.4)	180.15	8.20	676.74	451.16	1127.90
	(2.8, 3.0, 3.2)	179.70	8.20	675.33	450.22	1125.55
(32, 35, 38)	(2.0, 3.0, 4.0)	218.54	8.20	1736.75	1157.83	2894.58
	(2.2, 3.0, 3.8)	218.24	8.20	1734.20	1156.13	2890.33
	(2.4, 3.0, 3.6)	217.94	8.20	1731.66	1154.44	2886.10
	(2.6, 3.0, 3.4)	217.65	8.20	1729.13	1152.76	2881.89
	(2.8, 3.0, 3.2)	217.35	8.20	1726.61	1151.08	2877.69

**Table 6:** The return contract policies with $w = 20$

$\tilde{p}$	$\tilde{v}$	$q^{*}$	$b^{*}$	$E\left[ {\tilde{\varPi }_{R} } \right]^{*}$	$E\left[ {\tilde{\varPi }_{M} } \right]^{*}$	$E\left[ {\tilde{\varPi }_{SC} } \right]^{*}$
(22, 25, 28)	(2.0, 3.0, 4.0)	181.51	15.86	429.03	706.02	1135.05
	(2.2, 3.0, 3.8)	181.05	15.81	428.24	704.41	1132.65
	(2.4, 3.0, 3.6)	180.60	15.76	427.45	702.81	1130.26
	(2.6, 3.0, 3.4)	180.15	15.72	426.66	701.23	1127.90
	(2.8, 3.0, 3.2)	179.70	15.67	425.88	699.67	1125.55
(32, 35, 38)	(2.0, 3.0, 4.0)	218.54	11.84	2091.30	803.28	2894.58
	(2.2, 3.0, 3.8)	218.24	11.78	2089.22	801.11	2890.33
	(2.4, 3.0, 3.6)	217.94	11.72	2087.14	798.96	2886.10
	(2.6, 3.0, 3.4)	217.65	11.66	2085.06	796.83	2881.89
	(2.8, 3.0, 3.2)	217.35	11.60	2082.97	794.72	2877.69

8. The change of fuzziness of salvage value $\tilde{v}$ will not impact on the wholesale price $w^{*}$ in the RS contract. This is because the wholesale price $w^{*}$ is impacted only by the parameters $\tilde{c}_{s}$ and $\tilde{c}_{r}$. In addition, the return price $b^{*}$ decreases slightly as the fuzziness of salvage value $\tilde{v}$ decreases in the return contract.
9. When the fuzziness of salvage value decreases, the expected profits for the manufacturer and the retailer decrease. That is to say, the decreasing fuzziness of salvage value results in decreasing order quantity thereby the fuzzy expected profit of the retailer, the manufacturer and the whole supply chain decreases. Therefore, for the supply chain members, they should seek as high fuzziness of salvage value $\tilde{v}$ as possible in both contracts.

Based on the three discussions above, the following findings can be achieved:

10. Both contracts can achieve the coordination of the supply chain in a fuzzy environment, in which the manufacturer and the retailer obtain the same total fuzzy expected profits as the centralized decision-making system.
11. The manufacturer and the retailer will lower the fuzziness of demand, and increase the fuzziness of retail price and salvage value in order to deal with the environment uncertainty, which increases the retailer’s order quantity and eventually leads to the increase of the fuzzy expected profits.

## Conclusions

This article deals with the coordination problems of the retailer and the manufacturer in a fuzzy decision environment, where the RS contract and the return contract are employed. For examining the performance of supply chain members in two contracts, we apply the fuzzy set theory to solve these fuzzy models. We find that the change of fuzziness of the demand does not impact on the wholesale prices in both contracts, the supply chain members should seek as low fuzziness of demand, and high fuzziness of retail price and salvage value as possible. The model proposed in this article is easy to perform and needs little data, and can be apply for newly development items with a short life cycle such as PC, communication and consumer electronic category.

One limitation in this study is that we only consider one retailer and one manufacturer in a two stage supply. Future research can be done for the situations including two or more competing supply chain members or in a multi-stage supply chain. The other limitation is that the parameters of the supply chain models are considered as triangular fuzzy numbers. In fact, the membership function of the fuzzy number can be nonlinear, one can consider the case the parameters are fuzzy random variables. The third limitation is that the supply chain members are all assumed to be risk neutral. It is still interesting to discuss the problem how to design the contract policies when the supply chain members are risk averse or risk preference in a fuzzy environment.
